# Involvement of autophagy in mesaconitine-induced neurotoxicity in HT22 cells revealed through integrated transcriptomic, proteomic, and m6A epitranscriptomic profiling

**DOI:** 10.3389/fphar.2024.1393717

**Published:** 2024-06-13

**Authors:** Xiaohuang Lin, Jian Zhang, Zekai Wu, Yuan Shi, Mengting Chen, Maodong Li, Hong Hu, Kun Tian, Xiaoqi Lv, Chutao Li, Yang Liu, Xinyue Gao, Qiaomei Yang, Kunqi Chen, An Zhu

**Affiliations:** ^1^ Key Laboratory of Ministry of Education for Gastrointestinal Cancer, School of Basic Medical Sciences, Fujian Medical University, Fuzhou, China; ^2^ Department of Preventive Medicine, School of Public Health, Fujian Medical University, Fuzhou, China; ^3^ State Key Laboratory of Mariculture Breeding, College of Marine Sciences, Fujian Agriculture and Forestry University, Fuzhou, China; ^4^ Shenzhen Bay Laboratory, Institute of Systems and Physical Biology, Shenzhen, China; ^5^ Key Laboratory of Cell Proliferation and Differentiation of the Ministry of Education, Peking University Genome Editing Research Center, College of Life Sciences, Peking University, Beijing, China; ^6^ Department of Gynecology, Fujian Maternity and Child Health Hospital (Fujian Obstetrics and Gynecology Hospital), Fuzhou, China

**Keywords:** mesaconitine, proteomic, autophagy, N6-methyladenosine modification, neurotoxicity

## Abstract

**Background:** Mesaconitine (MA), a diester-diterpenoid alkaloid extracted from the medicinal herb *Aconitum carmichaelii*, is commonly used to treat various diseases. Previous studies have indicated the potent toxicity of aconitum despite its pharmacological activities, with limited understanding of its effects on the nervous system and the underlying mechanisms.

**Methods:** HT22 cells and zebrafish were used to investigate the neurotoxic effects of MA both *in vitro* and *in vivo*, employing multi-omics techniques to explore the potential mechanisms of toxicity.

**Results:** Our results demonstrated that treatment with MA induces neurotoxicity in zebrafish and HT22 cells. Subsequent analysis revealed that MA induced oxidative stress, as well as structural and functional damage to mitochondria in HT22 cells, accompanied by an upregulation of mRNA and protein expression related to autophagic and lysosomal pathways. Furthermore, methylated RNA immunoprecipitation sequencing (MeRIP-seq) showed a correlation between the expression of autophagy-related genes and N6-methyladenosine (m6A) modification following MA treatment. In addition, we identified METTL14 as a potential regulator of m6A methylation in HT22 cells after exposure to MA.

**Conclusion:** Our study has contributed to a thorough mechanistic elucidation of the neurotoxic effects caused by MA, and has provided valuable insights for optimizing the rational utilization of traditional Chinese medicine formulations containing aconitum in clinical practice.

## 1 Introduction

*Aconitum* (*Aconitum carmichaelii*), an annual or perennial herb in the Ranunculaceae family, is a well-known traditional Chinese medicine used extensively for treating rheumatoid arthritis, cardiovascular diseases, and cancers (Zhou et al., 2015). However, *Aconitum* has been reported to have neurotoxic and cardiotoxic effects, which can lead to poisoning events (Chan, 2015). Accidental ingestion of *Aconitum* or consumption of herbal decoctions prepared from it may result in severe poisoning (Bello-Ramírez and Nava-Ocampo, 2004). Therefore, it is imperative to investigate the mechanisms of toxicity associated with *Aconitum* sp. alkaloids.

Mesaconitine (MA) is a diester–diterpenoid alkaloid found in *Aconite*, which is considered one of the most important bioactive and toxic constituents (Sun et al., 2014). MA exhibits a range of pharmacological activities, including vasorelaxation (Mitamura et al., 2002a), analgesic effects (Murayama et al., 1984), and anti-epileptiform effects (Ameri, 1998). Previous studies have demonstrated its analgesic effect in mice (Murayama and Hikino, 1985), its role in increasing the uptake of [3H] noradrenaline and neuronal excitability in rat hippocampal pyramidal cells (Ameri and Seitz, 1998), its elevation of intracellular Ca^2+^ concentration in human umbilical vein endothelial cells (HUVECs), and its induction of aortic relaxation via oxide production in rats (Ogura et al., 2004). Although various components of *Aconitum* possess analgesic effects, MA exhibits the most potent analgesic effects among these alkaloids by activating the noradrenergic and serotonergic descending systems (Murayama and Hikino, 1985; Zhao L. et al., 2020).

Due to the close proximity between therapeutic and toxic doses, the clinical treatment window of MA is narrow, resulting in frequent adverse reactions and toxic events during clinical use, which limits its widespread application. Animal studies have shown that a single oral administration of MA has a median lethal dose (LD_50_) of 1.9 mg/kg, while the intravenous LD_50_ value in mice was 0.068 mg/kg (Singhuber et al., 2009; Ye et al., 2012). Previous research on MA has primarily focused on its cardiac toxicity. MA exposure induces cardiac malformations, pericardial effusion, and cardiac arrest in zebrafish embryos (Ye et al., 2021). Additionally, it causes coagulation necrosis of the rat myocardium (Chen et al., 2023). Whole-cell patch-clamp recordings conducted on isolated guinea pig ventricular cardiomyocytes have demonstrated that MA treatment depolarizes the resting membrane potential and reduces action potential amplitude (Wang X. C. et al., 2021). However, the toxic effects of MA on the nervous system should not be neglected. An epidemiological study revealed that among the 41 patients who were hospitalized due to aconitine poisoning from 2008 to 2017, nervous system symptoms were most prevalent (21 cases) (Chung et al., 2021). However, the neurotoxicity of MA, especially its mechanism of action, remains poorly studied. Therefore, obtaining a more profound comprehension of its neurotoxicity mechanism is crucial for the advancement of novel *Aconitum* preparations, guiding the safer and more rational application of traditional Chinese medicine containing *Aconitum* in clinical practice, as well as evaluating its safety.

Previous studies have indicated a potential correlation between alkaloid treatment and RNA epigenetic modification (Li et al., 2021; Zhao et al., 2021). In recent years, approximately 160 RNA modifications have been documented, encompassing N1-methyladenosine (Dominissini et al., 2016), 5-methylcytosine (Ma et al., 2022), N6-methyladenosine (m6A), and 7-methylguanosine (Song et al., 2020). Among these modifications, m6A stands out as the most abundant and widespread in eukaryotes, constituting 0.1%–1.79% of the main base in mammals (Jones et al., 2020). RNA m6A modification has been reported to play a role in various biological processes, encompassing cell proliferation, differentiation, development, and immune response (Erson-Bensan and Begik, 2017). In the past few years, the advent of methylated RNA immunoprecipitation sequencing (MeRIP-seq) has enabled us to comprehensively delineate the landscape of RNA m6A modification and elucidate its biological functions (Meyer et al., 2012).

In this study, we proposed that MA induces adverse biological effects in HT22 mouse hippocampal neurons, including decreased cell viability, along with the elevated release of lactate dehydrogenase (LDH) and accumulation of oxidative stress. To elucidate the molecular mechanisms underlying MA-induced toxicity in HT22 cells, we performed high-throughput mRNA-seq and proteomics on MA-treated and untreated control cells at the same time point. We constructed a comprehensive profile of gene and protein expression associated with neurotoxicity caused by MA, thereby identifying autophagy as a specific signaling pathway regulating neurotoxicity. Furthermore, MeRIP-seq revealed that MA-induced autophagy is related to m6A modification. Additionally, we identified potential regulators of m6A methylation and the protein–protein interaction (PPI) network involved in MA-induced neurotoxicity. Notably, our findings demonstrate that MA inhibits neural development in zebrafish embryos *in vivo*, thus providing a novel model for the diagnosis and treatment of MA-induced neurotoxicity.

## 2 Materials and methods

### 2.1 Chemical reagent

**FIGURE 1:**
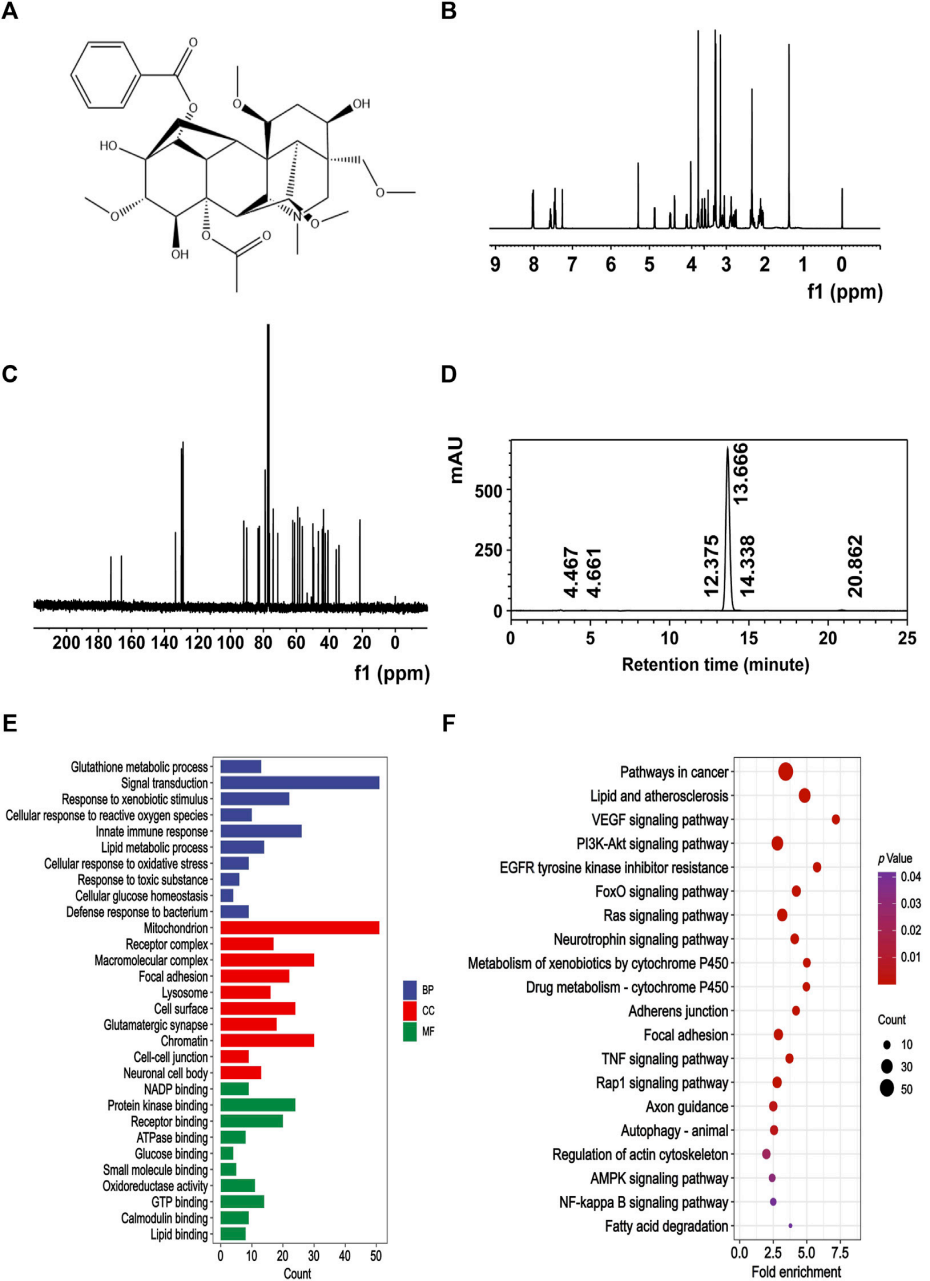
Chemical signature of MA. **(A)** Chemical structure of MA. **(B)** 400 MHz ^1^H NMR spectra of MA. **(C)** 100 MHz ^13^C NMR spectra of MA. **(D)** The purity of MA was quantified by HPLC. The retention time of MA was 13.666 min, and the area percent of MA was 98.979%. Representative GO **(E)** and KEGG **(F)** enrichment pathways of potential target proteins of MA.

MA was obtained from Must Bio-Technology Co., Ltd. The chemical structure of MA (Figure 1A) underwent confirmation through nuclear magnetic resonance (NMR) spectral analysis (Bruker) (Figures 1B, C), and the purity of MA was 98.979% (Figure 1D), as determined by high-performance liquid chromatography (HPLC) (Thermo Fisher Scientific Inc.).

### 2.2 Prediction of potential target proteins of MA in the PharmMapper server

The PharmMapper server was used to predict the potential target proteins of MA. First, the 2D structure of MA was obtained from the PubChem database and a mol2 file was generated using energy minimization principles. This file was then uploaded to the PharmMapper server. The UniProt database was utilized to standardize the UniProt IDs to gene symbols. The Gene Ontology (GO) and Kyoto Encyclopedia of Genes and Genomes (KEGG) pathways of the predicted targets were enriched using DAVID (National Institute of Allergy and Infectious Diseases, NIAID).

### 2.3 Cell culture and treatment

HT22 cell lines were originally obtained from the National Collection of Authenticated Cell Cultures. They were cultured in Dulbecco’s modified Eagle’s medium (Basal Media) containing 10% fetal bovine serum (Gibco) and then maintained in an incubator under 5% CO_2_ at 37°C. MA was dissolved in dimethyl sulfoxide (DMSO; Sangon), and the final working concentrations of MA were 0, 400, 800, and 1,600 μM.

### 2.4 Cell viability

HT22 cells at a density of 4 × 10^3^ cells per well in the logarithmic growth phase were plated on a 96-well plate and then exposed to 0, 400, 800, and 1,600 μM of MA for 72 h. Afterward, each well was supplemented with 10% CCK-8 solution (Yeasen) and incubated at 37°C for 2 h. Subsequently, the OD value at 450 nm was detected using a microplate reader (BioTek).

### 2.5 LDH release assay

LDH release in the cell supernatant was assessed following the manufacturer’s instructions after stimulation with 0, 400, 800, and 1,600 μM of MA for 72 h using an LDH assay kit (Beyotime). The OD value at 490 nm was determined using a microplate reader (BioTek).

### 2.6 Measurement of reactive oxygen species accumulation

The HT22 cells were plated in a 24-well plate at a density of 6 × 10^4^ cells per well, treated with 0, 400, 800, and 1,600 μM of MA for 72 h, and then incubated with 1 μM 2,7-dichlorofluorescein diacetate (DCFH-DA; Solarbio) in a dark environment at 37°C for 30 min. Reactive oxygen species (ROS) accumulation was assessed by measuring the dichlorofluorescein intensity using a fluorescence microscope (ZEISS).

### 2.7 Measurement of malondialdehyde content and superoxide dismutase activity

Protein concentration was determined using the BCA method. The levels of malondialdehyde (MDA) content and superoxide dismutase (SOD) activity in HT22 cells were measured after treatment with 0, 400, 800, and 1,600 μM of MA for 72 h using an MDA assay kit (Njjcbio) and an SOD activity assay kit (Njjcbio), respectively. The OD values were measured at 530 and 560 nm using a microplate reader (BioTek), respectively.

### 2.8 RNA preparation and high-throughput m6A MeRIP-seq

Given the results from the cell viability experiments, we selected the group treated with 800 μM MA with HT22 cell viability at approximately 70% for MeRIP-seq. The HT22 cells were plated in 6 cm dishes at a density of 2 × 10^6^ cells per dish, followed by exposure to 0 and 800 μM MA for 72 h before being lysed using TRIzol Reagent (Invitrogen). The enrichment and sequencing of m6A-modified RNA were carried out by Seqhealth Technology Co., Ltd. In brief, polyadenylated RNA was enriched by VAHTS mRNA Capture Beads (Vazyme) from 10 µg of total RNA. Subsequently, mRNA was fragmented into 100–200 nt fragments, with 10% of the RNA fragments saved as input and the remaining portion subjected to m6A immunoprecipitation with an anti-m6A antibody (Synaptic Systems). Finally, the m6A-modified mRNA fragments were purified using an RNA Clean and Concentrator Kit (Zymo Research) and were then utilized for library construction and sequenced on a NovaSeq 6000 platform (Illumina). The RNA-seq and MeRIP-seq data have been uploaded to the GEO database (ID: GSE261320).

### 2.9 Tandem mass tag-based proteomics analysis

#### 2.9.1 Protein extraction and tryptic digestion

Based on the experimental results of cell viability, we selected the group treated with 800 μM MA with HT22 cells for proteomics analysis. The HT22 cells were plated in 6 cm dishes at a density of 2 × 10^6^ cells per dish, followed by exposure to 0 and 800 μM MA for 72 h before being lysed utilizing a lysis buffer (100 mM triethylammonium bicarbonate, 1% SDS). The supernatant was obtained, and its protein concentration was determined using BCA assays. A measure of 150 ug of protein was extracted from each sample and reduced with 10 mM Tris(2-carboxyethyl) phosphine hydrochloride for 60 min at 55°C, after which the protein was alkylated with 37.5 mM iodoacetamide for 30 min at room temperature in the dark. Then, precooled acetone was added and allowed to stand at −20°C for 4 h. The precipitate was collected and air-dried. The dried precipitate was mixed with 200 mM triethylammonium bicarbonate buffer, and the mixture was scattered. Trypsin was added to the proteins at a ratio of 1:50 (w:w) and incubated at 37°C overnight.

#### 2.9.2 Tandem mass tag labeling

The peptides were labeled with a set of TMTpro isobaric tags (Cat#A44520, Thermo Fisher Scientific) following the manufacturer’s instructions. The samples were labeled as follows: CT_1, 126; CT_2, 127N; CT_3, 127C; MA_1, 128N; MA_2, 128C; and MA_3, 129N. The labeled peptides were mixed, desalted, and subjected to vacuum drying.

#### 2.9.3 HPLC fractionation and LC-MS/MS analysis

The labeled peptides underwent fractionation into 15 offline fractions using the High-pH Reversed-Phase Peptide Fractionation Kit (Thermo Fisher Scientific). For LC-MS/MS analysis, the pre-fractionated peptides were dissolved in 0.1% formic acid (solvent A) and loaded onto a reversed-phase analytical column (75 μm × 50 cm; Acclaim PepMap C18). The gradient consisted of a stepwise increase from 2% to 30% solvent B (0.1% formic acid in 80% acetonitrile) over 50 min, followed by a transition from 30% to 50% in 5 min, reaching 80% in 1 min, and then maintained at 80% for 4 min, all at a consistent flow rate of 300 nL/min in the EASY-nLC 1200 UHPLC System. The separated peptides were subjected to the NSI source, followed by tandem mass spectrometry (MS/MS) in the Orbitrap Exploris 480 MS. The spray voltage was set to 2.3 kV, and the heated capillary temperature was maintained at 320°C. MS1 spectra were acquired with a resolution of 60,000 FWHM, an AGC target of 300%, and a mass range of 350–1,600 m/z. The mass spectrometer was operated in a data-dependent mode. The top 12 precursor ions were fragmented by higher-energy collisional dissociation with a normalized collision energy of 34%. The MS/MS scan was set at a resolution of 30,000, with an AGC target of 200%, an isolation window of 0.7 m/z, injection times of 50 ms, and TurboTMT enabled. The intensity threshold was maintained at 2E4.

#### 2.9.4 Database searching of MS data

Mass spectrometric files were processed using Proteome Discoverer version 3.0. The data were searched against the *Mus musculus* UniProt database (17,192 sequences, downloaded in December 2023), assuming digestion with trypsin. TMTpro 16-plex based MS2 reporter ion quantification was selected. The reporter ion intensities were corrected using correction factors obtained from the reagent manufacturer’s certificate of analysis for TMTpro16 lot number XB341494. The variable modifications included the oxidation of methionine and acetylation of the protein N-terminus, while carbamidomethyl of cysteine and TMTpro of lysine and the N-terminus were specified as fixed modifications. The precursor ion mass error was set at 10 ppm, and the fragment ion mass error was set at 0.02 Da. The maximum number of missed cleavage sites allowed was 2, with a minimum peptide length of 7 and a maximum of 3 variable PTMs per peptide. Peptides and proteins were considered identified only if the false discovery rate was below 1%. The raw proteomic data have been deposited to ProteomeXchange (ID: PXD052001).

### 2.10 Bioinformatics analysis

The raw MeRIP-seq data were analyzed using fastp, followed by alignment to the mm10 genome reference sequences using HISAT2 (Kim et al., 2019). RNA expression levels and differential expression were analyzed using StringTie (Pertea et al., 2016) and DESeq2 (Love et al., 2014), respectively. The m6A peak calling and differential methylation detection were performed using exomePeak2 (Meng et al., 2013; Tang et al., 2021). The Poisson generalized linear model was used in exomePeak2 for estimating the methylation level and identifying differentially methylated regions, while sequencing depth size factors were estimated using exomePeak on non-methylated background regions. The m6A motif sequence was identified using STREME (Bailey, 2021) by integrating a position weight matrix Markov model into its algorithm. MetaTX was used for visualizing the distribution of epitranscriptome profiles (Wang Y. et al., 2021). GO and KEGG annotations were conducted using DAVID (Jiao et al., 2012). The m6A conservation and disease association data were obtained from ConsRM (Song et al., 2021) and RMDisease (Song et al., 2023a), respectively. The substrates of m6A regulators were identified using CLIP-seq datasets (Wang et al., 2020; Liu et al., 2022; Yin et al., 2022). The m6A patterns of gene methylated sites were visualized using Integrative Genomics Viewer (IGV) software. The PPI analysis was conducted using STRING, and the results were visualized using Cytoscape software. The gene set enrichment analysis (GSEA) and heatmaps were generated at https://www.bioinformatics.com.cn (last accessed on 20 February 2023).

### 2.11 Mitochondrial membrane potential detection

The mitochondrial membrane potential (MMP) was determined using the JC-10 probe (Solarbio). The JC-10 probe accumulates in the mitochondrial matrix, forming red fluorescent aggregates. Upon the decrease in MMP, the JC-10 probe diffuses out of the mitochondria, changes to monomeric form, and stains the cell with green fluorescence. The HT22 cells were plated in a 24-well plate at a density of 6 × 10^4^ cells per well and treated with 0, 400, 800, and 1,600 μM of MA for 72 h. Subsequently, the cells were incubated with the JC-10 probe at 37°C for 20 min. Finally, red and green fluorescence were analyzed using a microscope (ZEISS).

### 2.12 Determination of the mitochondrial permeability transition pore

The opening of the mitochondrial permeability transition pore (mPTP) was assessed using an mPTP Fluorescence Assay Kit (Beyotime) following the manufacturer’s instructions. Calcein AM enters the cell by passive transport and accumulates in the mitochondria. In cells, the barely non-fluorescent calcein AM is hydrolyzed by intracellular esterases to produce calcein, which has no membrane permeability, thereby retaining calcein in the cell and causing the cytoplasm to fluoresce in strong green. When calcein binds to Co^2+^, the fluorescence signal is quenched. Under normal conditions, the mPTP is closed, preventing Co^2+^ from entering the mitochondria. However, when mitochondria are damaged, the mPTP opens, allowing Co^2+^ to enter and bind with calcein, resulting in the quenching of green fluorescence.

Briefly, HT22 cells were exposed to 0, 400, 800, and 1,600 μM of MA for 72 h, followed by incubation with calcein AM staining solution for 45 min at 37°C. Subsequently, the staining solution was replaced with a cell culture medium, and incubation continued for another 30 min at 37°C. Finally, green fluorescence was analyzed using a microscope (ZEISS).

### 2.13 Determination of the intracellular Ca^2+^ level

The intracellular calcium ion concentration was measured using the Fluo-4 AM probe (Beyotime). Upon entering the cell, Fluo-4 AM was enzymatically cleaved by esterases to produce Fluo-4, which could bind to calcium ions and produce strong green fluorescence. Following the treatment of HT22 cells with 0, 400, 800, and 1,600 μM of MA for 72 h, the cells were exposed to a working solution of Fluo-4 AM at 37°C for 45 min. Following this, the cells underwent three washes with PBS and were subsequently incubated for an additional 20 min. The resulting green fluorescence was then analyzed using a microscope (ZEISS).

### 2.14 Detection of mitochondrial activity

Mitochondrial activity was detected using the MitoTracker Red CMXRos probe (Beyotime). The MitoTracker Red CMXRos probe contains mildly thiol-reactive chloromethyl that specifically marks biologically active mitochondria and emits red fluorescence. After a 72-h treatment with 0, 400, 800, and 1,600 μM of MA, the cells were exposed to MitoTracker Red CMXRos at 37°C for 15 min. Following the removal of the solution, a fresh cell culture medium was added, and the cells were analyzed using a fluorescence microscope (ZEISS).

### 2.15 Transmission electron microscopy

HT22 cells were plated in 6 cm dishes at a density of 2 × 10^6^ cells per dish and treated with 0, 400, 800, and 1,600 μM MA for 72 h. Afterward, the HT22 cells were prefixed in a mixture of 1.5% paraformaldehyde and 3% glutaraldehyde for 2 h at 4°C and in a mixture of 1% osmic acid and 1.5% potassium ferrocyanide for 1 h at 4°C. The samples were dehydrated in an ethanol gradient solution and embedded in epoxy resin for staining and sectioning. Ultrathin sections were observed using an EM 208 transmission electron microscope (Philips, Amsterdam, Netherlands).

### 2.16 RNA isolation and quantitative real-time polymerase chain reaction

Quantitative real-time polymerase chain reaction (qRT-PCR) was utilized to detect the expression of genes. After treating HT22 cells with 0, 400, 800, and 1,600 μM of MA for 72 h and treating zebrafish larvae with 0, 30, and 60 μM of MA at 1 day post-fertilization (dpf) for 2 days, TRIzol Reagent (Invitrogen) was used to extract total RNA. Subsequently, the extracted RNA was reverse-transcribed into cDNA. qRT-PCR was performed using a MonAmp ChemoHS qPCR Mix Kit (Mona) according to standard protocols. *Actb* served as an internal reference gene. All PCR primer sequences are shown in Table 1.

**TABLE 1:** Primer sequence of quantitative real-time polymerase chain reaction.

Gene	Primer sequence (5′ to 3′)
*Ctsb*-forward	CTC​ATG​TAG​GCT​GCT​TAC​CAT​A
*Ctsb*-reverse	TCT​CCT​TCA​CAC​TGT​TAG​ACA​C
*Ctsd*-forward	CAT​CTA​TCC​GTC​GGA​CTA​TGA​C
*Ctsd*-reverse	ATC​AAA​GAC​GAC​TGT​GAA​ACA​C
*Lamp1*-forward	CAG​CAC​TCT​TTG​AGG​TGA​AAA​A
*Lamp1*-reverse	GCC​ATT​TTT​CAG​TAC​TTC​TGC​A
*Lamp2*-forward	GTG​TGT​GAA​GAA​GAC​CAA​ACT​C
*Lamp2*-reverse	CTA​ATG​CTG​TAG​TTT​CCA​ACG​G
*Rab7*-forward	AAA​GAC​CTC​TCT​CAT​GAA​CCA​G
*Rab7*-reverse	CAG​TCA​CAT​CAA​ACA​CCA​GAA​C
*Tsc1*-forward	ATC​TTC​ATG​CCA​GTG​TTT​ATG​C
*Tsc1*-reverse	CAC​TTC​TTC​AAA​AGT​CTC​CAC​G
*Actb*-forward	CTA​CCT​CAT​GAA​GAT​CCT​GAC​C
*Actb*-reverse	CAC​AGC​TTC​TCT​TTG​ATG​TCA​C
*elavl3-*forward	CTA​TCA​ACA​CGC​TCA​ACG​GTC​TC
*elavl3-*reverse	GCT​CAC​ATA​CAG​GTT​GGC​ATC​G
*gap43-*forward	AAG​AGG​AGG​AGA​ACG​GAG​AAG​TG
*gap43-*reverse	GAG​TTA​GGC​TGC​TCT​GGT​TTG​G
*gfap-*forward	GCA​GGA​GAC​TGA​GGA​GTG​GTA​TC
*gfap-*reverse	GAA​TCT​GTC​GGC​GAT​AGT​CAT​TGG
*mbpa -*forward	ATC​AGC​AGG​TTC​TTC​GGA​GGA​G
*mbpa-*reverse	GAC​TTA​GGA​CGA​GGA​GAG​GAC​AC
*actb-*forward	CCC​AAA​CCC​AAG​TTC​AGC​CA
*actb-*reverse	ACC​CAC​GAT​GGA​TGG​GAA​GA

Note: *Ctsb*, cathepsin B; *Ctsd*, cathepsin D; *Lamp1*, lysosomal-associated membrane protein 1; *Lamp2*, lysosomal-associated membrane protein 2; *Rab7*, member RAS oncogene family; *Tsc1*, TSC complex subunit 1; elavl3, ELAV-like RNA-binding protein 3; *gap43*, growth-associated protein 43; *gfap*, glial fibrillary acidic protein; *mbpa*, myelin basic protein a; *Actb*, actin beta.

### 2.17 Western blotting

HT22 cells were plated in a 6-well plate at a density of 5 × 10^5^ cells per well and treated with 0, 400, 800, and 1,600 μM MA for 72 h. Total protein was extracted by RIPA lysate containing phosphatase inhibitors and proteinase inhibitors (Beyotime). The proteins were separated by sodium dodecyl sulfate–polyacrylamide gel electrophoresis (SDS-PAGE) and then transferred onto PVDF membranes and blocked with 5% skimmed milk. Then, the membranes were incubated with primary antibodies for PINK1, LC-3B, p62, and β-actin (Proteintech) overnight at 4°C. Next, the membranes underwent three washes with TBST buffer and were co-incubated with secondary antibodies for 1 h. Finally, the membrane proteins were exposed to ECL ultra-high-sensitivity luminescent liquid, and images were obtained using an Amersham Imager 680 System (Cytiva).

### 2.18 Molecular docking

The interaction between MA and m6A-modified differentially expressed regulators was investigated through molecular docking using SYBYL-X 2.0 software. The 3D structures of proteins were obtained from AlphaFold (Jumper et al., 2021) and pre-treated using SYBYL-X 2.0 to add hydrogen atoms, remove heteroatoms and water molecules, and repair side chains (Wu et al., 2022b). The 2D structure of MA was obtained from the PubChem compound database and subsequently transformed into 3D structures based on energy minimization principles (Zhao J. et al., 2020). The total docking score, which comprehensively evaluated solvation, entropy, hydrophobic complementarity, and polar complementarity, was considered indicative of a stable interaction between the proteins and molecules when it exceeded 5 (Zhu et al., 2019).

### 2.19 Molecular dynamics simulation

To simulate the protein–ligand interaction, we utilized the Simulation Package tOward Next GEneration (Huang et al., 2022). The FF14SB united-atom force field (Maier et al., 2015) was used consistently throughout the simulation study. The complex systems were solved using the SPC/E water model (Berendsen et al., 1987) in cubic boxes, ensuring a minimum distance of 1.2 nm from the cube edge. Potassium and chloride ions were added to neutralize the systems. For each system, molecular dynamics (MD) runs lasting 50 ns were executed. To evaluate binding stability in a dynamic environment, various MD trajectory analyses, including root mean square deviation (RMSD), root mean square fluctuation (RMSF), radius of gyration (Rg), H-bond occupancy, and total binding free energy, were applied to analyze the simulation results.

### 2.20 Cellular thermal shift assay

HT22 cells were treated with 0 and 800 μM MA in 6-cm dishes for 72 h. After washing the cells once with PBS, 350 μL of PBS was added to collect and re-suspend the cells. The cell suspension was divided into aliquots, with each tube containing 50 μL. The metal-bath temperature was set at 37, 41, 45, 49, 53, 57, and 61°C for heating the individual tubes for a duration of 3 min. Following heat treatment, the cells underwent three cycles of freeze–thawing using liquid nitrogen and were subsequently centrifuged at 12,000 ×*g* for 10 min. Following centrifugation, protein samples were prepared using the supernatant, and the interaction between METTL14 and MA was confirmed by Western blot.

### 2.21 Zebrafish husbandry and treatment

Zebrafish (AB line) were raised at 28.5°C under a 14-h light/10-h dark cycle in a circulating system, and all procedures were conducted in compliance with the regulations of the Institutional Animal Care and Use Committee (IACUC) of Fujian Medical University. The Fujian Medical University IACUC reference number was FJMU2023-Y-0824. The zebrafish larvae were treated with 30 and 60 μM MA at 1 dpf for 2 days. Siblings were treated with 0.1% DMSO as a control. The survival rate and hatching rate were calculated at 2 days post-treatment (dpt), and the body length was measured at 1 dpt and 2 dpt, respectively, to evaluate the influence of MA treatment on zebrafish development. Zebrafish were anesthetized using a 0.02% tricaine solution before imaging.

### 2.22 Zebrafish locomotor behavior tracking

Wild-type zebrafish larvae were exposed to 5 μM MA or 0.1% DMSO at 4 dpf, and locomotor behavior was assessed at 9 h post-treatment (hpt) using Noldus EthoVision^®^ XT video tracking software (version 10.1.856; Noldus Information Technology, Netherlands) and the Noldus DanioVision^®^ zebrafish tracking hardware system. Zebrafish larvae were placed in 12-well plates (one larva per well) at 28°C and acclimated for 10 min. The behavior of zebrafish larvae was then recorded for 5 min, followed by the analysis of the locomotor distance, duration, speed, and meander.

### 2.23 Statistical analysis

The experimental data were analyzed using SPSS and presented as the mean ± standard deviation. A one-way analysis of variance was used for group comparison, with a significance level set at *p* < 0.05.

## 3 Results

### 3.1 GO and KEGG enrichment of the predicted target proteins of MA

We first used the PharmMapper server to predict the potential target proteins associated with MA and found 302 potential target proteins. In order to gain further insights into their functional characteristics, GO and KEGG enrichment analysis were conducted using DAVID. This analysis resulted in the identification of 471 significant GO entries and 142 significant KEGG entries, with a statistical level set at *p* < 0.05. The GO enrichment terms mainly included mitochondrion, lysosome, and oxidoreductase activity (Figure 1E). The KEGG enrichment terms mainly included drug metabolism-cytochrome P450, adherens junction, and autophagy-animal (Figure 1F).

### 3.2 MA-induced neurotoxicity *in vitro* and *in vivo*

**FIGURE 2:**
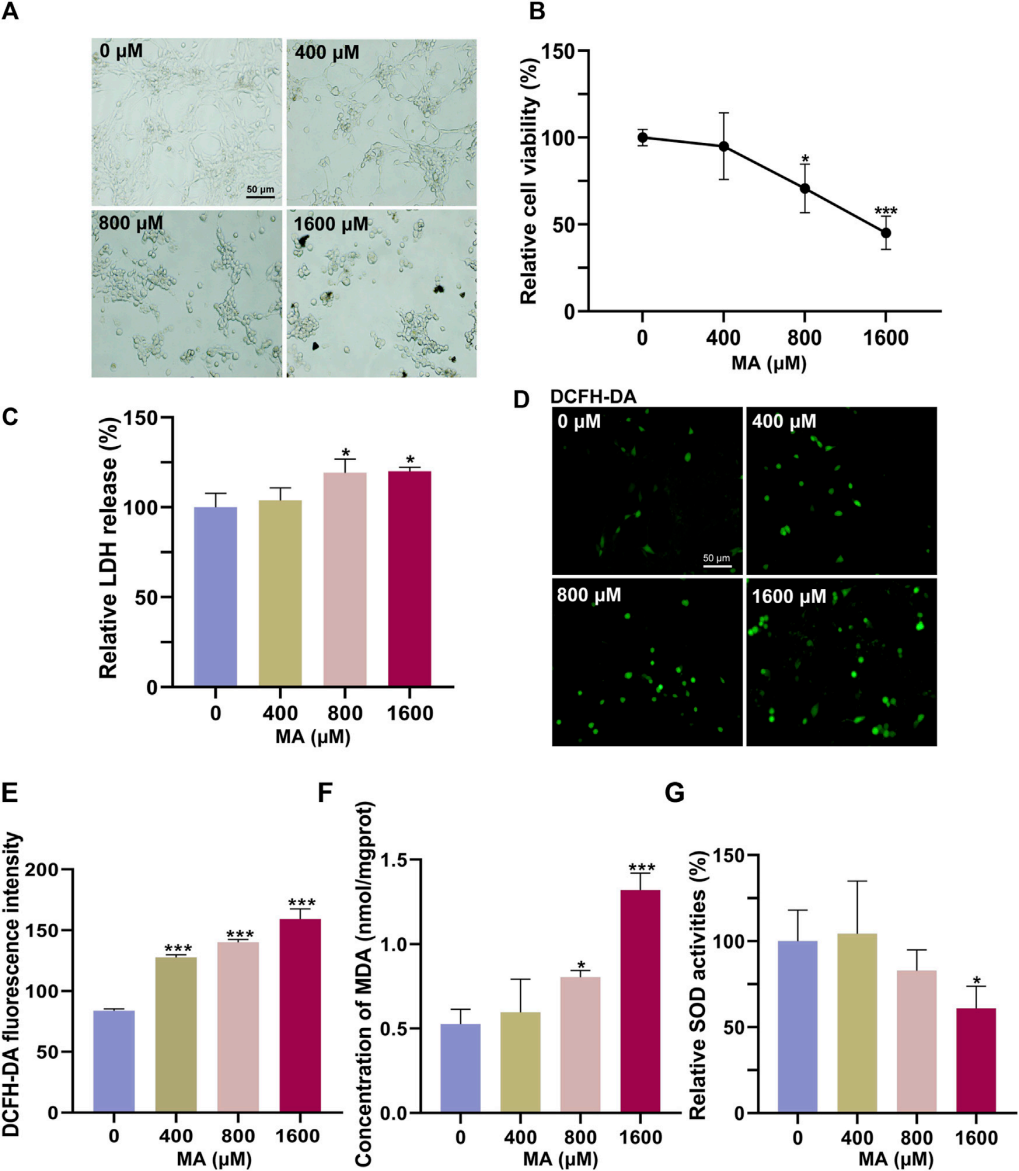
Cytotoxic effects of MA on HT22 cells. **(A)** Representative images of HT22 cells treated with MA for 72 h. **(B)** CCK-8 results showing the cytotoxic effects of MA on HT22 cells for 72 h. **(C)** Quantitative results of relative LDH levels, normalized to the untreated group. **(D)** DCFH-DA staining result showing ROS generation after MA treatment. **(E)** Statistical analysis of **(D)** showing that DCFH-DA fluorescence was significantly increased after 72 h of MA treatment. **(F)** MDA content was measured after treatment with MA. **(G)** Quantitative results of relative SOD activities, normalized to the untreated group. *p < 0.05, **p < 0.01, and ***p < 0.001 compared with the control group.

We first used HT22 cells to examine the neurotoxic effects of MA *in vitro*. HT22 cells were treated with different concentrations of MA for 72 h. In the control group, HT22 cells exhibited a polygonal shape with well-defined boundaries (Figure 2A). The cell body demonstrated extensive branching growth and interconnectedness, forming a complex network structure. Following exposure to MA, the cellular morphology became rounder, more oval-shaped, and heteromorphic. Additionally, the interconnection of cell processes was reduced, and the network structure became less apparent. The CCK-8 results showed that the viability of HT22 cells decreased to 70.67% (*p* < 0.05) and 45.11% (*p* < 0.001) after treatment with 800 and 1,600 μM MA for 72 h, respectively (Figure 2B). The release of LDH was detected following MA treatment, revealing a dose-dependent induction of LDH release from MA-treated cells (*p* < 0.05), thereby indicating the cytotoxicity of MA toward HT22 cells (Figure 2C).

ROS are byproducts generated during normal cellular metabolism. Under physiological conditions, intracellular antioxidant systems predominantly eliminate ROS to maintain their low levels. However, when the intracellular antioxidant system is inhibited or there is an imbalance between the antioxidant system and the production of ROS, oxidative stress occurs. DCFH-DA staining results showed significant ROS accumulation after treatment with 400, 800, and 1,600 μM MA (*p* < 0.001), indicating that MA induces oxidative stress in HT22 cells (Figures 2D, E). Furthermore, the MA-induced oxidative damage was confirmed by an increase in the lipid peroxidation marker MDA, accompanied by a significant decrease in the activity of the antioxidant enzyme SOD (Figures 2F, G).

**FIGURE 3:**
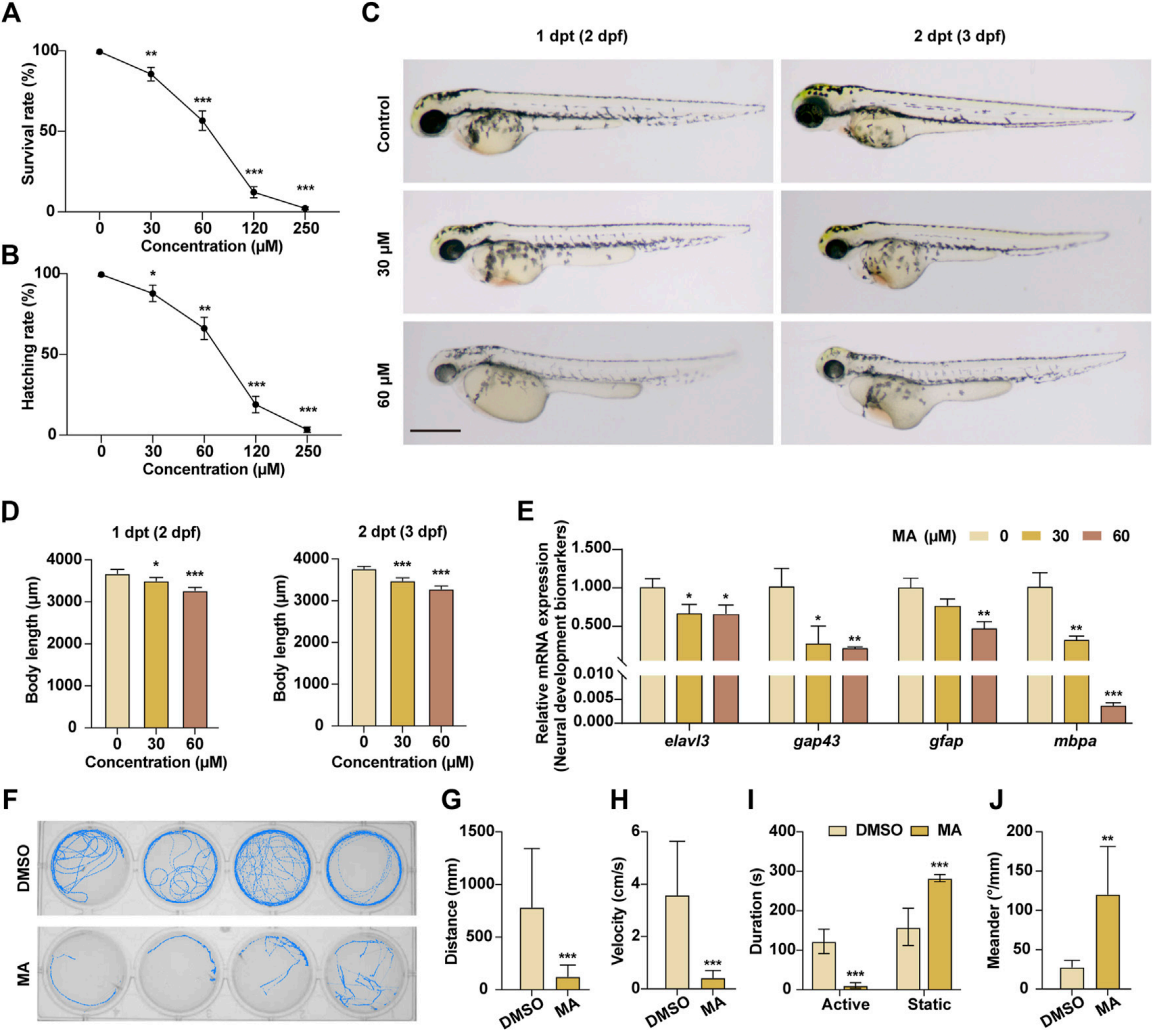
MA inhibited neural development of zebrafish embryos *in vivo*. **(A)** Survival rate of zebrafish embryos at 2 dpt. **(B)** Hatching rate of zebrafish embryos at 2 dpt. **(C)** Embryo morphology of control and MA-treated zebrafish embryos at 1 dpt and 2 dpt. Scale bar: 200 μm. **(D)** Quantification results of the body length of control and MA-treated zebrafish embryos at 1 dpt and 2 dpt. **(E)** qRT-PCR results of neural development-related genes of MA-treated zebrafish embryos at 1 dpf for 2 days. **(F)** Locomotor behavior of zebrafish larvae was measured after treatment with MA. **(G–J)** Statistic result of locomotor distance **(G)**, velocity **(H)**, movement duration **(I)**, and meander **(J)**. *p < 0.05, **p < 0.01, and ***p < 0.001 compared with the control group.

The process and specific mechanism of neurogenesis in zebrafish closely resemble those observed in mammals. Consequently, zebrafish serves as a valuable vertebrate model for investigating and assessing the neurotoxicity of drugs. In order to elucidate the *in vivo* neurotoxic effects of MA, we exposed 1-dpf zebrafish embryos to varying concentrations of MA. We observed a significant dose-dependent decrease in both survival and hatching rates at 2 dpt with MA (Figures 3A, B). Furthermore, pericardial edema and cardiac congestion were observed following MA treatment, consistent with previous reports of cardiotoxicity. Notably, surviving zebrafish exhibited a small-head phenotype and significantly reduced body length (Figures 3C, D). After treating zebrafish larvae with 0, 30, and 60 μM of MA at 1 dpf for 2 days, the mRNA expression levels related to neural development were measured. As shown in Figure 3E, MA treatment significantly decreased the mRNA expression levels of genes associated with neurodevelopment, such as *elavl3*, *gap43*, *gfap*, and *mbpa*. In order to investigate the potential interference of MA on the neuromotor system of zebrafish, we conducted behavioral analyses following MA treatment. The administration of MA resulted in a significant reduction in locomotor distance, movement duration, and speed (Figures 3F–I). Furthermore, larvae exposed to MA exhibited twitching movements characterized by enhanced meandering behavior, indicative of neuronal damage-related motor impairments (Figure 3J). The above results indicated that MA treatment inhibited neural development in zebrafish.

### 3.3 Differential gene expression in MA-treated HT22 cells

**FIGURE 4:**
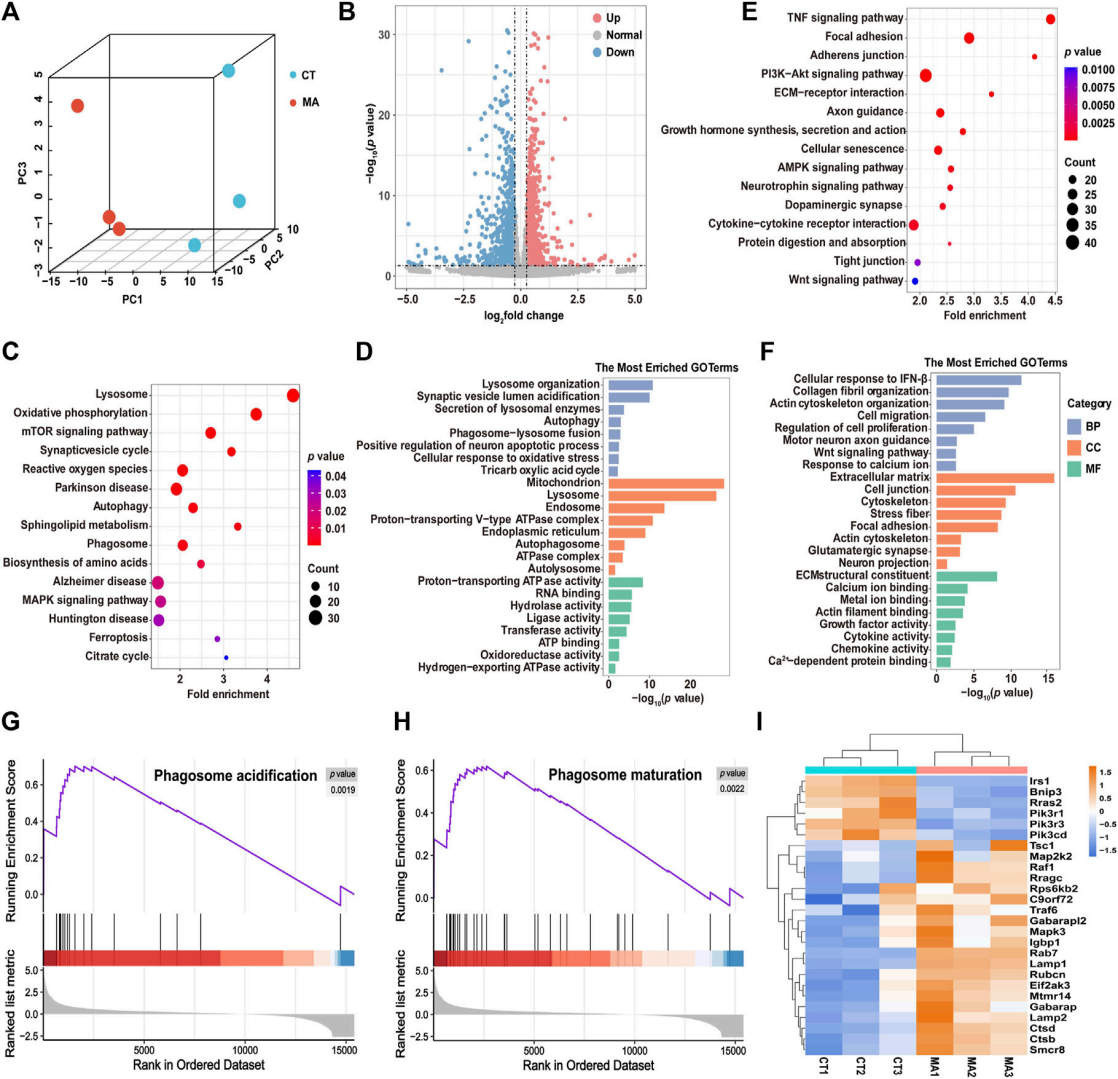
Molecular diversity of HT22 cells after MA treatment. **(A)** PCA showing the clustering of HT22 cells into two subclusters, control (CT) and MA-treated (MA) cells. **(B)** Volcano plot showing DEGs between control and MA-treated cells. **(C,D)** Representative KEGG **(C)** and GO **(D)** enrichment pathways of upregulated genes after MA treatment. **(E,F)** Representative KEGG **(E)** and GO **(F)** enrichment pathways of downregulated genes after MA treatment. **(G,H)** GSEA of **(G)** phagosome acidification and **(H)** phagosome maturation and the difference between control and MA treated cells. **(I)** Heatmap showing scaled expression levels of DEGs involved in autophagy between control and MA-treated cells.

To explore the neurotoxicological mechanism of MA, we performed RNA-seq analysis on HT22 cells with or without MA treatment. Given the results from the cell viability experiments, we selected the group treated with 800 μM MA with HT22 cell viability at approximately 70% for mRNA-seq. Principal component analysis (PCA) showed that the cells treated with MA were segregated into distinct groups compared to the untreated cells, indicating a significant alteration in the cellular state following MA treatment (Figure 4A). A total of 2,291 differentially expressed genes (DEGs) were identified, comprising 1,126 upregulated and 1,165 downregulated genes after MA treatment (Figure 4B).

DAVID was utilized to perform KEGG and GO pathway analysis in order to predict the associated signaling pathways and biological functions related to the DEGs. The KEGG pathway analysis showed that the upregulated genes were mainly enriched in the lysosome, the mTOR signaling pathway, and autophagy (Figure 4C). GO enrichment analysis was categorized into three groups: biological process (BP), cellular component (CC), and molecular function (MF). The most enriched BP terms were associated with lysosome organization, autophagy, and phagosome–lysosome fusion (Figure 4D). CC terms were involved in the mitochondrion, proton-transporting v-type ATPase complex, and autophagosomes. The MF terms exhibited significant enrichment in proton-transporting ATPase activity, RNA binding, and oxidoreductase activity. In addition, the downregulated genes after MA treatment were found to be involved in several KEGG pathways, including the TNF signaling pathway, focal adhesion, PI3K-Akt signaling pathway, and cellular senescence (Figure 4E). The most enriched BP terms were associated with cell migration, regulation of cell proliferation, motor neuron axon guidance, and response to calcium ions. CC terms were enriched in the extracellular matrix, cell junction, and cytoskeleton. MF terms were involved in calcium ion binding, metal ion binding, and actin filament binding (Figure 4F).

Next, we performed GSEA to further elucidate the signaling pathways associated with MA treatment. Consistently, phagosome acidification and phagosome maturation were upregulated after MA treatment compared to control cells (Figures 4G, H). Additionally, RNA-seq data revealed that the expression level of autophagy-associated genes was upregulated in MA-treated HT22 cells, suggesting that MA induced autophagy in mouse neurons (Figure 4I).

### 3.4 Proteomic analysis of MA-treated HT22 cells

**FIGURE 5:**
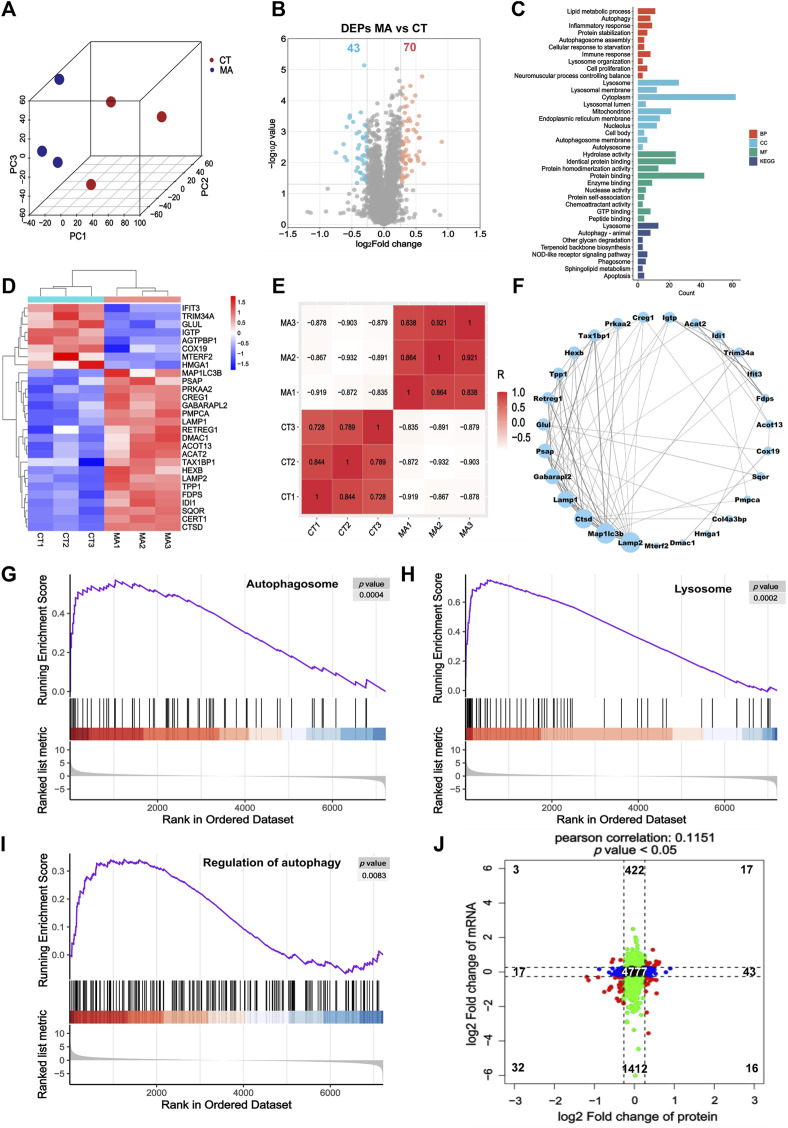
Proteomic analysis indicated that autophagy may be the potential pathway for MA-induced neurotoxicity. **(A)** PCA for the protein expression in different groups. **(B)** Volcano plot showing DEPs between control and MA-treated cells. **(C)** GO and KEGG pathway enrichment of the DEPs between control and MA-treated cells. **(D)** Heatmap showing changed expression levels of DEPs involved in the mitochondrion, autophagy, autophagosome assembly, lysosome organization, and autolysosome between control and MA-treated cells. **(E)** Correlation of DEPs in the mitochondrion, autophagy, autophagosome assembly, lysosome organization, and autolysosome between control and MA-treated groups. **(F)** Protein–protein interaction network of the mitochondrion, autophagosome assembly, lysosome organization, autolysosome, and autophagy. Compared to the control group, the GSEA of **(G)** the autophagosome, **(H)** lysosome, and **(I)** regulation of autophagy in the MA-treated group indicated that MA activated autophagy. **(J)** Scatter plot of 9-quadrant associate analyses of mRNA and proteins.

In order to investigate the effects of MA treatment on protein expression, we carried out a quantitative proteomic analysis to elucidate comprehensive protein changes in HT22 cells with or without MA treatment. The PCA revealed a distinct segregation of MA-treated cells compared to the control cells, aligning with our RNA-seq findings (Figure 5A). Volcano plots from the proteomic analysis displayed a total of 113 differentially expressed proteins (DEPs) in the MA-treated group, with 70 upregulated and 43 downregulated proteins relative to the untreated control (Figure 5B). GO and KEGG pathway enrichment analysis showed that DEPs induced by MA treatment were mainly enriched in biological processes, including the mitochondrion, autophagosome assembly, lysosome organization, autolysosome, and autophagy (Figure 5C). The heatmap illustrates the expression patterns of DEPs associated with the aforementioned terms (Figure 5D).

The correlation among biological replicates within the same group was high, with the absolute values of R minima higher than 0.7 and *p* < 0.05, which validated the reliability of our model and the reproducibility of the sample processing approach (Figure 5E). Subsequently, we examined the regulatory relationship among these genes using STRING to construct a PPI network. MAP1LC3B, LAMP2, CTSD, LAMP1, and GABARAPL2 emerged as the top five proteins, exhibiting degrees of 13, 13, 12, 10, and 9, respectively (Figure 5F). GSEA results indicated an upregulation in the regulation of autophagy, autophagosomes, and lysosomes following MA treatment, aligning with our RNA-seq findings (Figures 5G–I).

Furthermore, we examined the concordance in the directions of change between mRNA and protein expression levels (Figure 5J). The correlation between the mRNA and protein (*r* = 0.1151 and *p* < 0.05) was positive and highly significant. We identified genes that exhibited concordant increases in both mRNA and protein (*n* = 17), discordant patterns with mRNA decreasing and protein increasing (*n* = 16), discordant patterns with mRNA increasing and protein decreasing (*n* = 3), and concordant decreases in both mRNA and protein (*n* = 32). A total of 19 mRNAs and proteins showed inconsistent trends, which may be attributed to RNA post-transcriptional modification and post-translational processing and modification. Genes that overlap between mRNA-seq and proteomic analysis are listed in Supplementary Table S1.

### 3.5 MA treatment induced mitochondrial damage and triggered autophagy in HT22 cells

**FIGURE 6:**
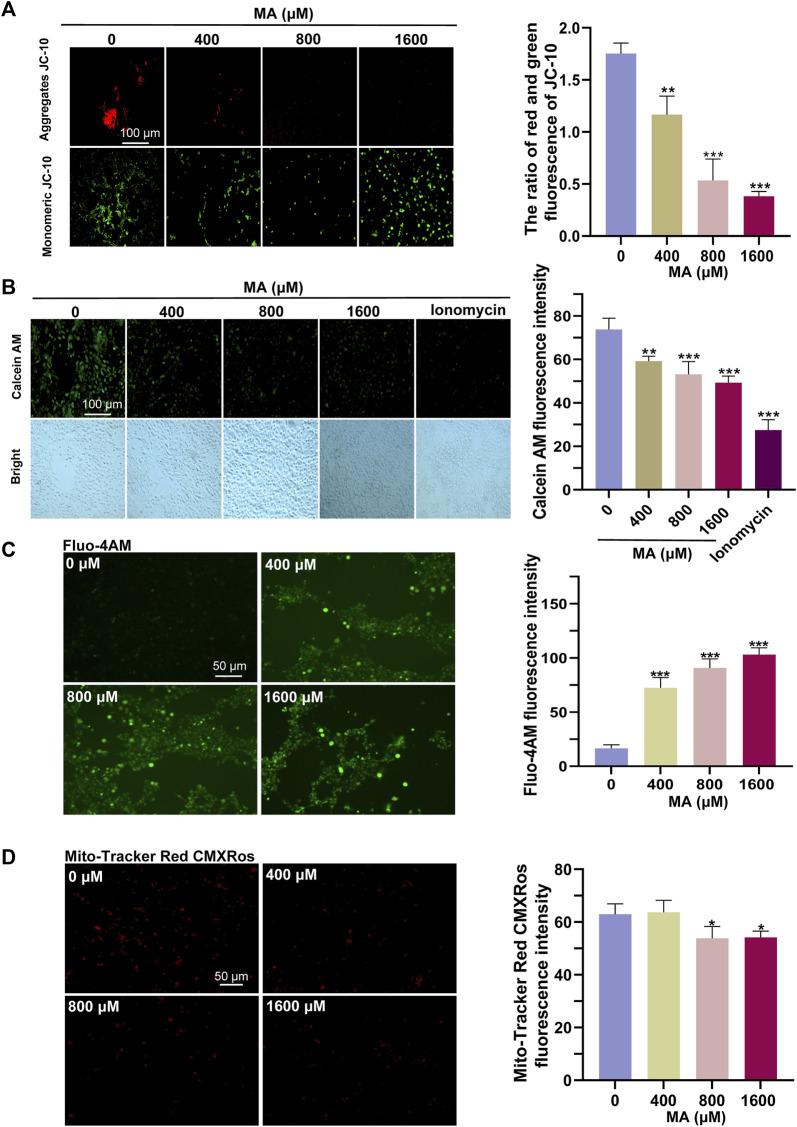
Detrimental effects of MA treatment on the mitochondria. **(A)** Representative images and quantitative results of the JC-10 probe indicated a significant reduction in MMP in MA-treated HT22 cells. **(B)** Representative images and quantitative results of the calcein AM probe indicated sustained opening of the mPTP in HT22 cells following MA treatment. **(C)** Representative images and quantitative results of the Fluo-4 AM probe showed an increase in intracellular Ca^2+^ levels in MA-treated HT22 cells. **(D)** Representative images and quantitative results of the MitoTracker Red CMXRos probe revealed a decrease in mitochondrial activity in MA-treated HT22 cells.

The results of RNA-seq and proteomics showed that MA exposure can affect the mitochondria. Therefore, we examined the functional status of the mitochondria. In our assessment of mitochondrial activities post-MA treatment, we observed that aggregates of JC-10, indicative of healthy and polarized mitochondria, decreased significantly, while monomeric JC-10, which signified membrane depolarization and dysfunction, increased (Figure 6A). Concurrently, the fluorescence of accumulated calcein in the mitochondria decreased, suggesting that MA treatment induced damage to the mPTP of HT22 cells (Figure 6B). The elevation of cytoplasmic calcium concentrations occurs through either the influx of extracellular calcium ions or the release of calcium ions from cellular organelles. This increase in cytoplasmic calcium levels facilitates the increased binding of intracellular Fluo-4 AM to calcium ions, resulting in a subsequent augmentation of green fluorescence intensity within the cell. Our findings indicate a dose-dependent increase in Fluo-4 AM fluorescence intensity post-MA treatment, reflecting an augmentation of mitochondrial calcium efflux (Figure 6C). In line with these observations, the fluorescence intensities of MitoTracker Red CMXRos showed a significant decrease, suggesting compromised mitochondrial activities in HT22 cells after MA treatment (Figure 6D).

**FIGURE 7:**
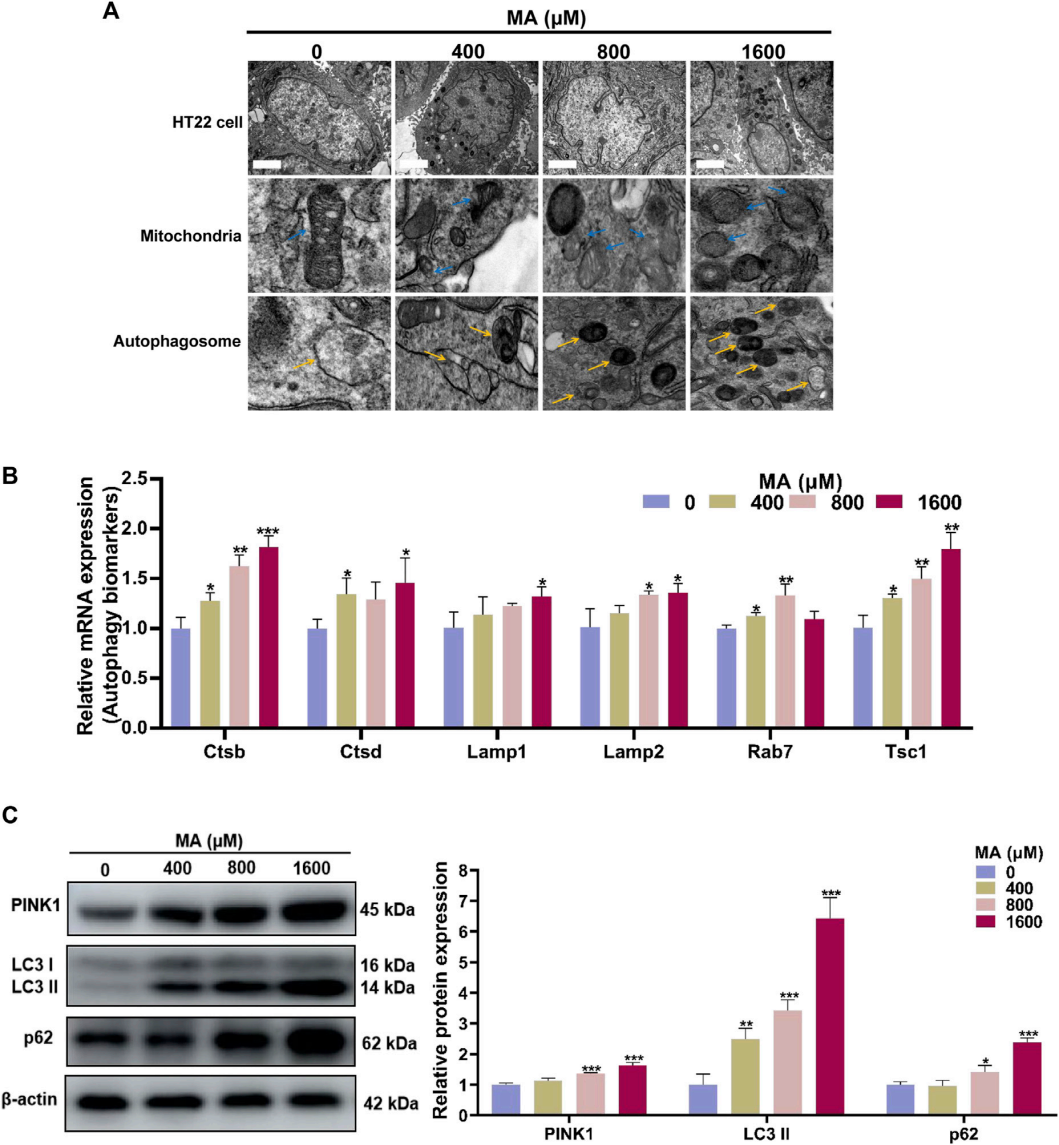
Autophagy was activated after MA treatment. **(A)** TEM images of the mitochondria and autophagosome in HT22 cells treated with MA. Blue arrows indicate mitochondria. Yellow arrows indicate the autophagosome. Scale bar: 2 μm. **(B)** qRT-PCR results of several components of autophagy signaling pathway in control and MA-treated cells. **(C)** Western blot analysis was performed to examine the levels of PINK1, LC3, and p62 in HT22 cells treated with MA. β-Actin was used as a loading control. *p < 0.05, **p < 0.01, and ***p < 0.001 compared with the control group.

Mitochondrial damage serves as a pivotal trigger for the induction of autophagy, which is a widely presented degradation/recycling system in eukaryotic cells and plays a crucial role in maintaining neuronal homeostasis. When stimulated, misfolded proteins or abnormal cell components are transported to the lysosome for degradation to maintain cellular self-renewal. In the TEM examination, the control group exhibited normal mitochondrial ultrastructure in HT22 cells. However, exposure to MA resulted in the dissolution of the mitochondrial membrane, the disappearance of mitochondrial cristae, and abnormalities in mitochondrial morphology across all MA-exposed groups. Autophagosomes, indicated by yellow arrows, are double-membrane vesicles formed during autophagy. They phagocytose a variety of intracellular substances and transport them to lysosomes. Subsequently, autophagosome–lysosome fusion occurs to generate autolysosomes for the degradation of these substances. Furthermore, there was an increase in the formation of autophagosomes and autolysosomes following the exposure of HT22 cells to 400, 800, and 1,600 μM MA (Figure 7A).

Through qRT-PCR analysis, we observed elevated expression levels of multiple genes involved in the autophagy pathways after MA treatment (Figure 7B). Consistently, Western blot analysis demonstrated a significant increase in the protein levels of PINK1, LC3-II, and p62 in HT22 cells exposed to 800 and 1,600 μM MA (*p* < 0.05) (Figure 7C). The above results demonstrated that MA exposure leads to mitochondrial damage and triggers autophagy in HT22 cells, playing a crucial role in the neurotoxicity of MA.

### 3.6 m6A modification pattern of MA-treated HT22 cells by transcriptome-wide MeRIP-seq

**FIGURE 8:**
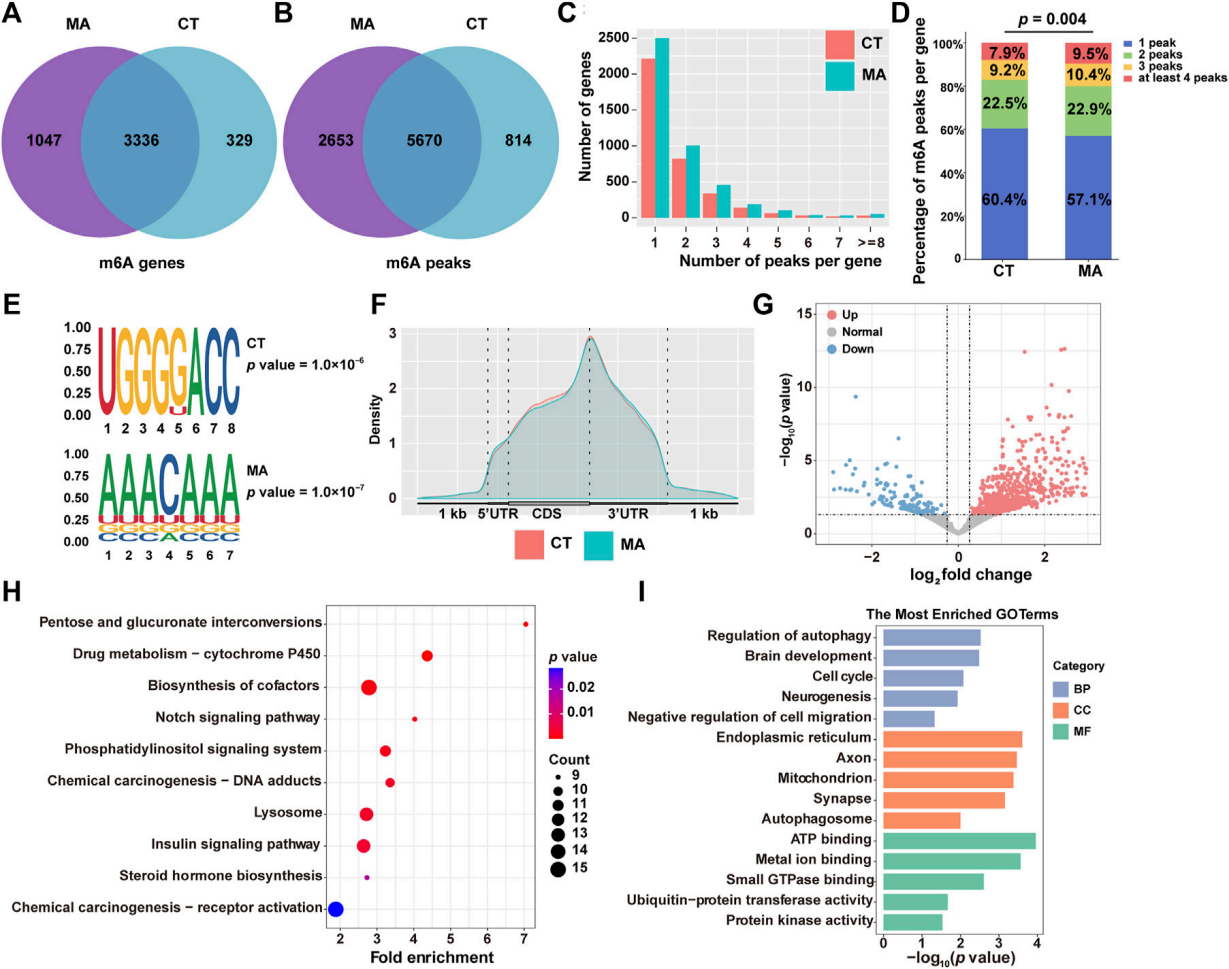
Modification patterns of m6A in MA-treated HT22 cells. **(A,B)** Venn diagram showing m6A-modified genes **(A)** and peaks **(B)** from control and MA-treated cells. **(C)** Number of m6A modification peaks per gene. **(D)** Chi-square test on the constituent ratio of the number of m6A-modified peaks in the control and MA groups. **(E)** STREME analysis of enriched motifs in m6A modification peak regions. **(F)** Density of m6A modification peaks in mRNA transcripts. **(G)** Volcano plot showing differentially m6A-modified genes between control and MA-treated cells. Representative KEGG **(H)** and GO **(I)** enrichment pathways of differentially m6A-modified genes after MA treatment.

RNA epigenetic modifications, particularly m6A, have been reported to play an essential role in the regulation of gene expression. To investigate whether m6A modification is involved in neurotoxicity following MA treatment, we performed MeRIP-seq on both MA-treated and untreated control cells. After stringent filtration, a total of 9,137 m6A peaks containing transcripts from 4,712 genes were identified. Specifically, 6,484 peaks corresponding to 3,665 genes were detected in control groups, and 8,323 peaks were identified after MA treatment, representing transcripts of 4,383 genes (Figures 8A, B).

Moreover, transcripts with m6A modifications were categorized according to the number of modification peaks present in each transcript. Statistical results revealed that each group had over 3,000 genes containing 1–2 m6A modification peaks, with a relatively small number of transcripts containing more than 4 m6A modification peaks (Figure 8C). Additionally, a chi-square test was conducted to examine the composition ratio of the number of m6A-modified peaks between the control and MA groups. The results indicated a significant difference in the composition ratio of the number of m6A-modified peaks between the two groups (Figure 8D). The conserved m6A sequence of RRACH (R represents purine and H represents a non-guanine base) was determined using STREME analysis, revealing the presence of classical m6A motifs in both control and MA-treated groups (Figure 8E). To determine the distribution pattern of m6A modification across the entire transcriptome, the density of m6A modification peaks in both the control and MA-treated groups was examined. The result suggested that m6A modification was enriched in the 5′ untranslated region (5′UTR), coding sequence (CDS), and 3′UTR (Figure 8F). Notably, the density of the m6A modification increased significantly in the 5′UTR and CDS regions, reaching its peak at the stop codon before rapidly decreasing in the 3′UTR.

Subsequently, we examined the differentially m6A-modified genes after MA treatment. Specifically, 616 genes exhibited increased m6A modifications, while 153 genes displayed weakened m6A modifications (Figure 8G). The KEGG pathway analysis revealed that these differentially modified genes were predominantly enriched in the cytochrome P450, Notch signaling pathway, and lysosome (Figure 8H). The most enriched BP terms were associated with the regulation of autophagy, brain development, cell cycle, and negative regulation of cell migration. CC terms were enriched in the endoplasmic reticulum, axon, mitochondrion, and autophagosome. MF terms were found to be involved in ATP binding, metal ion binding, and ubiquitin–protein transferase activity (Figure 8I). These annotation results were consistent with our transcriptomic and proteomic data, indicating that MA induces neurotoxicity by regulating m6A modification of genes involved in autophagy and cell proliferation and migration signaling pathways.

### 3.7 Differential m6A modification and gene expression in MA-treated HT22 cells

**FIGURE 9:**
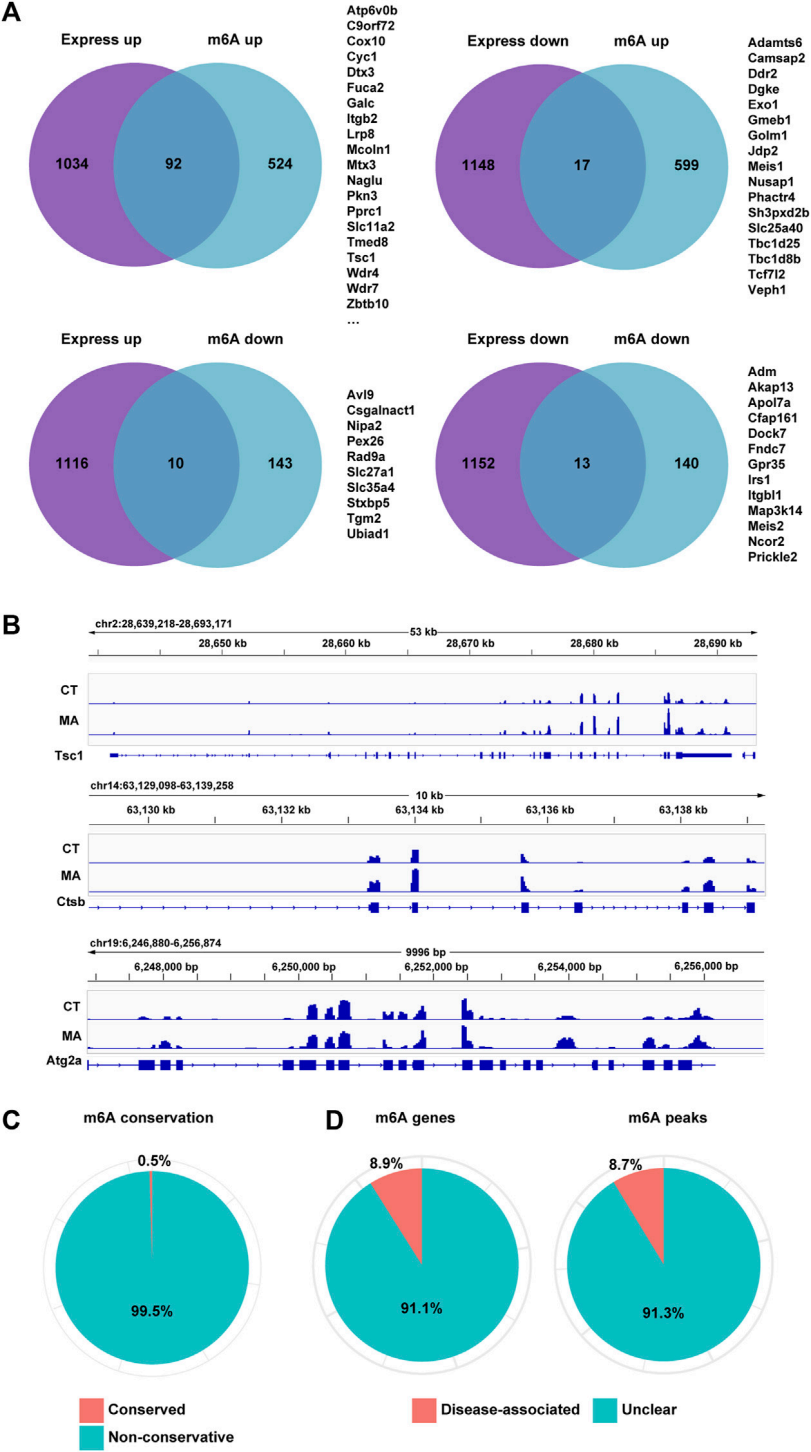
Differential m6A modification and gene expression in MA-treated HT22 cells. **(A)** Venn diagram showing differentially m6A-modified and expressed genes between control and MA-treated cells. **(B)** m6A modification levels on *Tsc1*, *Ctsb*, and *Atg2a* mRNA transcripts were observed using IGV. **(C,D)** Conservation of m6A sites **(C)** and disease association **(D)** of overlapped genes of differentially m6A-modified and expressed genes.

To further investigate the genes whose expression changes were regulated by m6A modification after MA treatment, we analyzed the overlapped genes with differential m6A modification and gene expression between control and MA-treated cells through MeRIP-seq and mRNA-seq data. The results showed that 92 genes and 17 genes were upregulated and downregulated after MA treatment, respectively, accompanied by enhanced m6A modification (Figure 9A). Additionally, 10 genes were upregulated and 13 genes were downregulated, accompanied by a decrease in m6A modification. The level of m6A modification on mRNA transcripts was visualized using IGV. Notably, autophagy-related genes *Tsc1*, *Ctsb*, and *Atg2a* exhibited elevated m6A methylation after MA treatment, suggesting that the activation of autophagy genes might be caused by enhanced m6A modification (Figure 9B). In order to investigate m6A conservation and disease association, we performed ConsRM and RMDisease analyses, respectively. The ConsRM analysis result revealed that 99.5% of m6A-modified regions were non-conservative in differentially modified m6A and DEGs, suggesting that the dysregulation of non-conserved m6A sites was more likely to be associated with disease pathogenesis (Figure 9C). Furthermore, RMDisease analysis showed that 8.9% of m6A genes and 8.7% of m6A peaks were associated with diseases, suggesting a potential correlation between MA and diseases (Figure 9D).

### 3.8 Potential regulators of genes involved in autophagy, lysosomes, and adherens junctions

To identify potential regulators of m6A methylation in MA-based neurotoxicity, we analyzed the expression levels of 22 previously reported m6A methylation writers, readers, and erasers. The results revealed that 6 m6A methylation regulators were significantly differentially expressed (*p* < 0.05), as shown in Table 2. Specifically, after MA treatment, writers VIRMA and METTL14 and reader YTHDF3 were upregulated, while writer RBM15B, reader IGF2BP2, and eraser ALKBH5 were downregulated. Meanwhile, MA treatment induced alterations in both gene expression levels and m6A methylation levels of genes related to the lysosome (*Fuca2*, *Galc*, *Mcoln1*, *Naglu*, *Slc11a2*, and *Wdr7*), autophagy (*C9orf72*, *Irs1*, and *Tsc1*), and adherens junction (*Tcf7l2*) (Figure 9A).

**TABLE 2:** mRNA expression levels of m6A regulators in MA-treated HT22 cells.

Genes	Regulation	Base mean	log_2_Fold change	*p*-value
*Virma*	Writer	4,809.56	0.2968	7.57 × 10^−4^
*Rbm15b*	Writer	3,028.51	−0.2055	3.29 × 10^−3^
*Alkbh5*	Eraser	9,731.20	−0.1402	7.34 × 10^−3^
*Mettl14*	Writer	3,032.21	0.1728	9.25 × 10^−3^
*Igf2bp2*	Reader	9,207.16	−0.2357	1.17 × 10^−2^
*Ythdf3*	Reader	3,333.01	0.3757	3.20 × 10^−2^
*Mettl5*	Writer	628.19	−0.2470	5.39 × 10^−2^
*Zc3h13*	Writer	5,768.37	0.2248	6.96 × 10^−2^
*Igf2bp1*	Reader	2,516.10	0.1337	9.35 × 10^−2^
*Rbm15*	Writer	1,678.79	0.1591	2.27 × 10^−1^
*Fmr1*	Reader	9,442.51	−0.1090	2.75 × 10^−1^
*Ythdf2*	Reader	1,908.00	0.0957	2.95 × 10^−1^
*Cbll1*	Writer	522.16	0.1285	3.16 × 10^−1^
*Hnrnpa2b1*	Reader	34,674.16	0.0972	3.29 × 10^−1^
*Fto*	Eraser	3,415.63	0.0556	4.33 × 10^−1^
*Ythdc1*	Reader	9,807.00	0.0779	4.76 × 10^−1^
*Wtap*	Writer	2,397.51	0.0484	5.78 × 10^−1^
*Hnrnpc*	Reader	13,644.78	−0.0301	5.96 × 10^−1^
*Ythdf1*	Reader	2,036.78	0.0307	6.72 × 10^−1^
*Mettl3*	Writer	615.61	−0.0361	7.71 × 10^−1^
*Ythdc2*	Reader	1,547.71	0.0249	7.72 × 10^−1^
*Igf2bp3*	Reader	4,029.10	−0.0079	9.83 × 10^−1^

**FIGURE 10:**
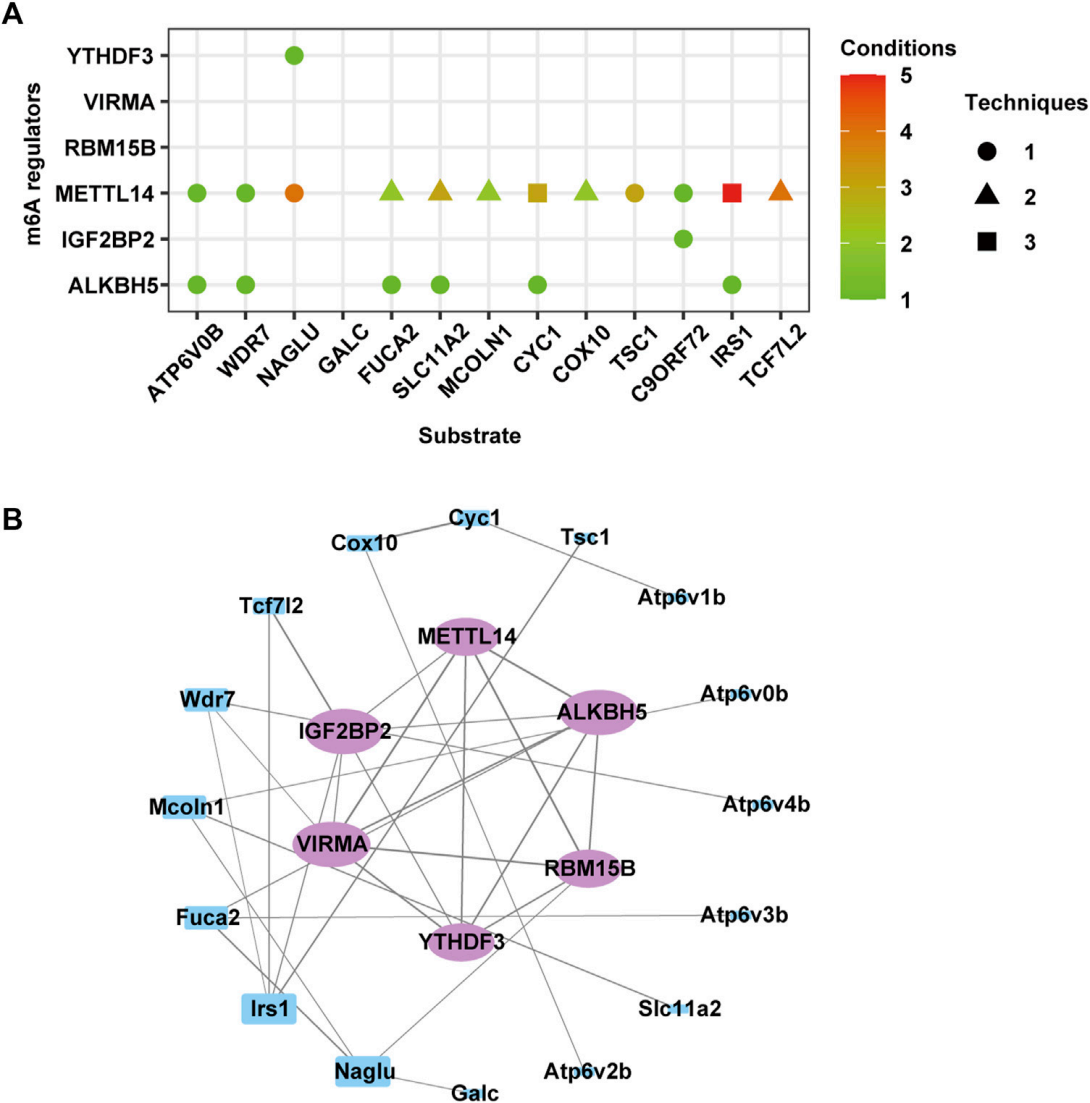
Predicted effect of m6A regulators. **(A)** Effect of m6A regulators on the overlapped genes of differentially expressed and m6A-modified genes involved in autophagy, lysosomes, and adherens junction. **(B)** Protein–protein interaction network of m6A regulators (purple circle) and genes in **(A)** (blue rectangle).

The CLIP-seq datasets were used to identify potential substrates of m6A methylation regulators, and the results suggested that METTL14 and ALKBH5 were broadly involved in regulating most genes in these four terms (Figure 10A). The readers YTHDF3 and IGF2BP2 specifically recognized m6A methylation on *Naglu* and *C9orf72*, respectively. To further explore the regulatory relationships between differentially expressed m6A regulators and these target genes, we used STRING to construct a PPI network. The top five genes, namely, *Irs1*, *Fuca2*, *Mcoln1*, *Naglu*, and *Wdr7*, exhibited degrees of 4, 3, 3, 3, and 3, respectively, suggesting the network regulation of m6A modification during neuronal damage caused by MA (Figure 10B).

### 3.9 Molecular interactions of MA with differentially expressed m6A modification regulators

**FIGURE 11:**
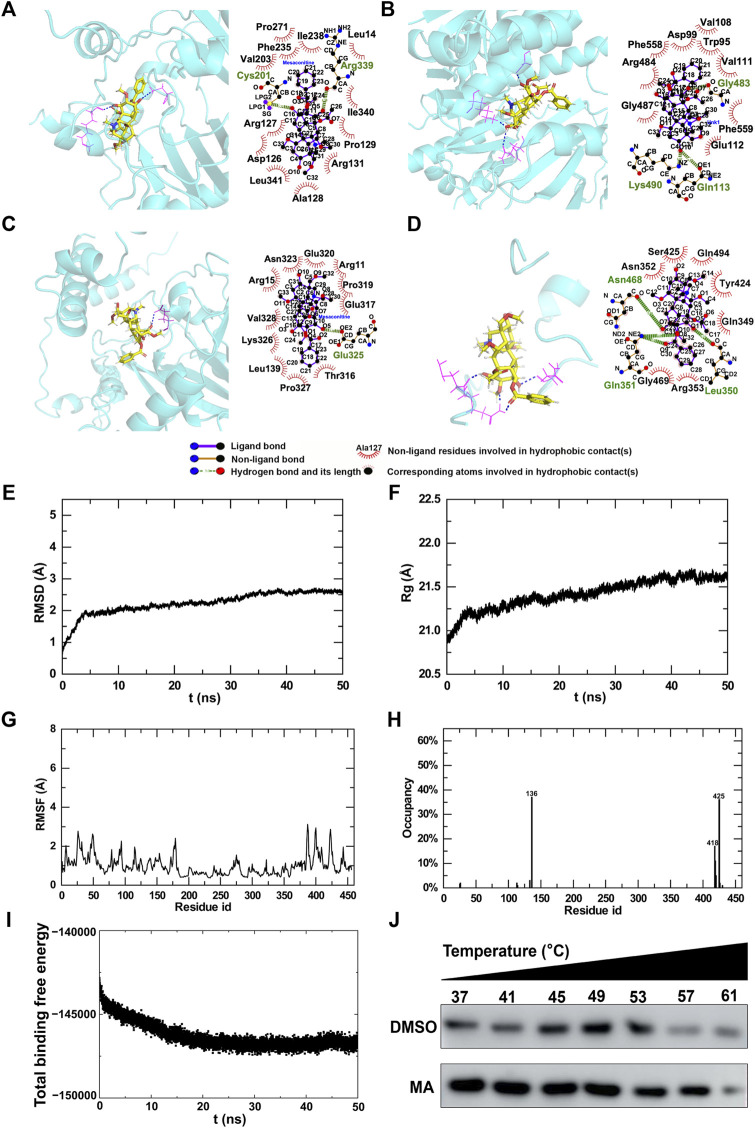
Molecular interactions between MA and m6A regulators. **(A–D)** 3D and 2D interaction models of ALKBH5 **(A)**, IGF2BP2 **(B)**, METTL14 **(C)**, and YTHDF3 **(D)**. Trajectory analysis of the MA-METTL14 complex during 50-ns MD simulations. **(E)** RMSD of the backbone atoms and **(F)** Rg of the complex versus time for the simulation. **(G)** RMSF and **(H)** H-bond occupancy of amino acids for complexes. **(I)** Energy changes in the MA-METTL14 complex during 50-ns MD simulations. **(J)** CETSA to verify MA binding to METTL14.

In order to explore the potential targets of MA, we assessed the molecular interactions between MA and differentially expressed m6A modification regulators, including ALKBH5, IGF2BP2, METTL14, and YTHDF3. All total scores were above 5, indicating stabilized interactions between MA and these m6A modification regulators (Table 3). Theoretically, MA bids ALKBH5 through the formation of a hydrogen bond at Cys201 and Arg399, as well as 12 hydrophobic contacts with Pro271, Phe355, Val203, lle238, Leu14, lle340, Pro129, Arg131, Ala128, Leu341, Asp126, and Arg127 (Figure 11A). MA binds IGF2BP2 through the formation of a hydrogen bond at Gln113, Lys490, and Gly483, as well as nine hydrophobic contacts with Val108, Asp99, Trp95, Val111, Phe558, Arg484, Gly487, Phe559, and Glu112 (Figure 11B). MA bound to METTL14 through the formation of a hydrogen bond at Glu317, as well as 11 hydrophobic contacts with Glu317, Pro319, Arg11, Glu320, Asn323, Arg15, Val328, Lys326, Leu139, Pro327, and Thr316 (Figure 11C). MA bids YTHDF3 through the formation of two hydrogen bonds at Gln351 and one hydrogen bond at Leu350 and Asn468, as well as seven hydrophobic contacts with Ser425, Asn352, Gln494, Tyr424, Gln349, Arg353, and Gly469 (Figure 11D). These results indicated that ALKBH5, IGF2BP2, METTL14, and YTHDF3 were potential targets of MA.

**TABLE 3:** Molecular interactions between MA and m6A regulators.

Protein	AlphaFold ID	Total score	Crash	Polar	H-bond number	Residues involved in H-bond formation	Hydrophobic contact number	Residues involved in hydrophobic contacts
ALKBH5	Q3TSG4	5.160	−3.955	1.867	2	Cys201 and Arg399	12	Pro271, Phe355, Val203, lle238, Leu14, lle340, Pro129, Arg131, Ala128, Leu341, Asp126, and Arg127
IGF2BP2	Q5SFO7	7.854	−3.035	4.284	3	Gln113, Lys490, and Gly483	9	Val108, Asp99, Trp95, Val111, Phe558, Arg484, Gly487, Phe559, and Glu112
YTHDF3	Q8BYK6	6.010	−2.876	2.20	4	Gln351 (two H-bonds), Leu350, and Asn468	7	Ser425, Asn352, Gln494, Tyr424, Gln349, Arg353, and Gly469
METTL14	Q3UIK4	5.552	−2.053	2.265	1	Glu317	11	Glu317, Pro319, Arg11, Glu320, Asn323, Arg15, Val328, Lys326, Leu139, Pro327, and Thr316

### 3.10 MD simulation and validation

The substrate prediction results showed that the m6A regulator METTL14 was the substrate of most genes (Figure 10A), and the molecular docking results demonstrated a stable interaction between MA and METT14 (Table 3). Therefore, based on these findings, we conducted molecular dynamics research on METTL14. In order to evaluate the binding stability and conformational variability of the MA-METTL14 complex during the MD simulation, we performed RMSD, Rg, and RMSF analyses. The average RMSD values of the complex ranged from 0.5 to 2.7 Å, while the Rg values exhibited fluctuations within 1 Å (Figures 11E, F). The RMSF analysis quantifies residue-specific fluctuations. During the simulation, none of the amino acid residues displayed conformational variations exceeding 3.2 Å relative to their average structure, indicating remarkable stability among backbone atoms in this complex system (Figure 11G). In the 456 amino acid residues, aspartic acid at position 136 demonstrated the highest hydrogen bond occupancy at 36% (Figure 11H). During the MD simulation, the binding free energy of the MA-METTL14 complex gradually decreased, suggesting a more stable binding state between MA and the METTL14 protein (Figure 11I). Molecular docking and MD simulations suggested that METTL14 could be a potential target of MA, which we then verified using the cellular thermal shift assay (CETSA). After treatment with 800 μM MA, the degradation rate of METTL14 in HT22 cells exhibited a deceleration trend with increasing temperature, suggesting that MA treatment enhanced the thermal stability of METTL14 (Figure 11J). The CETSA result suggested that MA binds to METTL14, in accordance with the predictions made by molecular docking and MD simulations.

## 4 Discussion

MA is a predominant and representative constituent of alkaloids contained in *Aconitum* (Sun et al., 2014), which is widely utilized for the treatment of various neurological diseases (Zhao L. et al., 2020). Previous studies have shown that MA possesses analgesic properties (Hikino and Murayama, 1985), acts as a vasodilator (Mitamura et al., 2002b), exhibits antiepileptic effects (Ameri, 1998), and displays antidepressant activity (Nesterova et al., 2011). However, it should be noted that MA is also a highly toxic component, necessitating the careful dosage administration of *Aconitum*, primarily containing MA and aconitine. *Aconitum* is widely recognized for its lethal neurotoxicity, with research primarily focused on aconitine and limited reports on MA-induced central nervous system toxicity (Chung et al., 2021).

A measure of 60 μM of aconitine was found to impede neuromuscular conduction in isolated diaphragm muscle by inhibiting the release of neurotransmitters, nerve action potentials, end-plate potentials, and indirect muscle action potentials (Onur et al., 1995). In addition, an aconitine-induced neuromuscular blockade was observed at concentrations of 2 μM, which resulted in the depression of twitch responses to nerve stimulation while having no effect on direct muscle contractions, indicating that aconitine induces a neuromuscular blockade by blocking the nerve compound action potential (Muroi et al., 1990). Given the structural similarity between aconitine and MA, we hypothesized that MA may also possess neurotoxic properties. In recent years, there has been extensive discussion on the cardiotoxicity of MA. Studies have shown that the treatment of HBEC-5i microvascular endothelial cells with MA resulted in a significant reduction in cell viability by perturbing Ca^2+^ signaling (Hsu and Liang, 2021). Furthermore, MA stimulated Ca^2+^ influx via the Na^+^/Ca^2+^ exchangers in HUVECs and induced aorta dilatation (Ogura et al., 2004). However, there has been insufficient investigation into the neurotoxic effects of MA, and its underlying mechanism remains unclear. Therefore, it is crucial to focus on the neurotoxicity induced by MA. In this study, HT22 cells derived from mouse hippocampal neurons were exposed to MA, and the changes in mRNA expression levels and m6A modifications were evaluated using RNA-seq and MeRIP-seq, respectively.

Oxidative stress, characterized by an imbalance between ROS production and the cellular antioxidant defense system, often serves as a catalyst for mitochondrial structure and function damage (Zorov et al., 2014; van Hameren et al., 2019). Mitochondrial damage, in turn, becomes a pivotal trigger for the activation of autophagy, a cellular mechanism designed to maintain balance and eliminate compromised cellular components (Zong et al., 2023). Moreover, oxidative regulations impact all stages of autophagy, encompassing induction, phagophore nucleation, phagophore expansion, autophagosome maturation, cargo delivery to the lysosome, and, ultimately, the degradation of cargo and recycling of products, alongside the transcription of autophagy genes (Zhou et al., 2022). In our study, we discovered that cells subjected to MA treatment exhibited an elevation in ROS levels, accompanied by a concurrent mitochondrial permeability transition and enhanced autophagy. This observation implies that MA may induce damage to HT22 cells by promoting an excess of ROS, resulting in mitochondrial dysfunction. Furthermore, these alterations likely act as initiators of autophagy in response to the cellular stress induced by MA. After treating HT22 cells with gradient concentrations of MA, an increased accumulation of ROS was observed. Previous studies have demonstrated that oxidative stress can induce autophagy, where ROS acts as the primary intracellular signal transducer, sustaining this process (Filomeni et al., 2015). As a lysosome-mediated self-digestion mechanism, autophagy plays a crucial role in preventing tissue damage by eliminating misfolded proteins and damaged organelles (Ye et al., 2022). The progression of autophagosome precursors involves a sequence of processes such as expansion, elongation, and nucleation, leading to the formation of double-layered spherical autophagosomes (Liu et al., 2020). Simultaneously, ATG12 and LC3 play regulatory roles in the expansion and closure of the autophagosome. Assisted by ATG3 and ATG7 proteins, autophagosomes undergo fusion with lysosomes, resulting in the formation of autolysosomes, whose contents are subsequently degraded (Shan et al., 2018). We hypothesized that MA treatment produces a large amount of ROS, which leads to cellular oxidative damage. Intracellular ROS accumulation served as the molecular initiating event of MA-induced neurotoxicity, subsequently leading to perturbed cell morphology, decreased cell viability, increased LDH release, and activation of autophagy.

MeRIP-seq, which combines RNA–protein immunoprecipitation with high-throughput sequencing technology, facilitates the analysis of m6A modification levels in RNA. These modifications play a crucial role in regulating various aspects of RNA metabolism, such as maturation, splicing, export, folding, translation, and stability, thus modulating the downstream signaling pathways and physiological functions (Wu et al., 2022a; Wu et al., 2022b; Zhang et al., 2023). The consensus motif sequence RRACH was identified in the m6A motif region through the analysis of MeRIP-seq data, which is consistent with previous findings (Dominissini et al., 2012). The majority of genes exhibited 1–3 peaks in the distribution of m6A modification, while some genes displayed a higher number of peaks, as previously demonstrated by Li et al. (2023). In addition, the CDS region contained the majority of m6A modification peaks, with the highest concentration observed around the stop codon, aligning with the topology of mouse RNA m6A methylomes (Dominissini et al., 2012). Furthermore, MA treatment induced 99.5% RNA m6A modification in un-conserved sites, and approximately 8.9% of genes were found to be associated with diseases, suggesting the potential correlation between this xenobiotic compound and neurological disorders. To investigate the impact of MA on biological function, we conducted GO and KEGG pathway enrichment analyses. The KEGG pathway analysis revealed the upregulation of the lysosome and autophagy following MA treatment. Conversely, the TNF signaling pathway, focal adhesion, and PI3K-Akt signaling pathway were found to be downregulated. Similarly, GO enrichment analysis showed enhanced lysosome organization, autophagy induction, and proton-transporting ATPase activity. Additionally, there was a decrease in cell migration, cell proliferation, and regulation of the extracellular matrix. These results demonstrated that the neurotoxicity of MA is tightly regulated by synergistic interactions among various signaling pathways.

The m6A modification regulates a range of biological processes, including transcription, pre-mRNA splicing, mRNA export, mRNA stability, and translation (Chen et al., 2019; Shi et al., 2019; Huang et al., 2020; Song et al., 2023b). RNA m6A modification is mediated by m6A methyltransferase, m6A demethylase, and m6A binding proteins. The biological effects of m6A methylation modifications depend on the recognition and binding of m6A-binding proteins (Zhao Y. et al., 2020). In this study, we further identified the regulators of m6A methylation for differentially expressed genes related to autophagy, lysosomes, and adherens junctions. A total of six differential expression levels were observed among the identified m6A regulators. Among them, ALKBH5, IGF2BP2, METTL14, and YTHDF3 were identified as potential substrates of m6A methylation regulators. Specifically, METTL14, an m6A reader, demonstrated the ability to catalyze m6A modification and exhibited the broadest involvement in regulating a majority of genes within these four terms. Molecular docking results revealed that MA directly bound with METTL14, with total scores higher than 5, suggesting that MA induces neurotoxicity by directly interacting with METTL14 to regulate m6A modification of autophagy-related genes. In this study, the mRNA expression level of autophagy-related gene *Tsc1* was significantly upregulated, and its m6A methylation level increased after MA treatment, accompanied by enhanced binding to METTL14.

## 5 Conclusion

Overall, our study provides a comprehensive analysis of MA-induced neurotoxicity, thereby providing valuable clues for optimizing the safety assessment of traditional Chinese medicine containing *Aconitum* in clinical practice as well as promoting its rational application.

## Figures and Tables

**Table:** 

**BP**	Biological process
**CC**	Cellular component
**CDS**	Coding sequence
**CETSA**	Cellular thermal shift assay
**CT**	Control
**DCFH-DA**	2,7-Dichlorofluorescein diacetate
**dpf**	Day post-fertilization
**dpt**	Day post-treatment
**DEGs**	Differentially expressed genes
**DEPs**	Differentially expressed proteins
**DMSO**	Dimethyl sulfoxide
**GO**	Gene Ontology
**GSEA**	Gene set enrichment analysis
**HPLC**	High-performance liquid chromatography
**IGV**	Integrative Genomics Viewer
**KEGG**	Kyoto Encyclopedia of Genes and Genomes
**LDH**	Lactate dehydrogenase
**MDA**	Malondialdehyde
**LD**_**50**_	Median lethal dose
**MA**	Mesaconitine
**MeRIP-seq**	Methylated RNA immunoprecipitation sequencing
**MMP**	Mitochondrial membrane potential
**mPTP**	Mitochondrial permeability transition pore
**MD**	Molecular dynamics
**MF**	Molecular function
**NMR**	Nuclear magnetic resonance
**PCA**	Principal component analysis
**PPI**	Protein–protein interaction
**qRT-PCR**	Quantitative real-time polymerase chain reaction
**Rg**	Radius of gyration
**ROS**	Reactive oxygen species
**RMSD**	Root mean square deviation
**RMSF**	Root mean square fluctuation
**SOD**	Superoxide dismutase
**TMT**	Tandem mass tag
**TEM**	Transmission electron microscopy
**UTR**	Untranslated region

## Data Availability

The original contributions presented in the study are publicly available. This data can be found at: GEO database, accession number GSE261320.

## References

[B1] Ameri A. (1998). Inhibition of stimulus-triggered and spontaneous epileptiform activity in rat hippocampal slices by the *Aconitum* alkaloid mesaconitine. Eur. J. Pharmacol. 342, 183–191. 10.1016/s0014-2999(97)01498-2 9548384

[B2] Ameri A. Seitz U. (1998). Effects of mesaconitine on [3H]noradrenaline uptake and neuronal excitability in rat hippocampus. Exp. Brain Res. 121, 451–456. 10.1007/s002210050480 9746152

[B3] Bailey T. L. (2021). STREME: accurate and versatile sequence motif discovery. Bioinformatics 37, 2834–2840. 10.1093/bioinformatics/btab203 33760053 PMC8479671

[B4] Bello-Ramírez A. M. Nava-Ocampo A. A. (2004). The local anesthetic activity of *Aconitum* alkaloids can be explained by their structural properties: a QSAR analysis. Fundam. Clin. Pharmacol. 18, 157–161. 10.1111/j.1472-8206.2004.00222.x 15066129

[B5] Berendsen H. J. C. Grigera J. R. Straatsma T. P. (1987). The missing term in effective pair potentials. J. Phys. Chem. 91, 6269–6271. 10.1021/j100308a038

[B6] Chan T. Y. (2015). Incidence and causes of *aconitum* alkaloid poisoning in Hong Kong from 1989 to 2010. Phytotherapy Res. PTR 29, 1107–1111. 10.1002/ptr.5370 25974837

[B7] Chen Q. Deng X. Zhang K. Kang Y. Jiao M. Zhang J. (2023). Changes to PUFA-PPAR pathway during mesaconitine induced myocardial coagulative necrosis. Food Chem. Toxicol. Int. J. Publ. Br. Industrial Biol. Res. Assoc. 177, 113831. 10.1016/j.fct.2023.113831 37182599

[B8] Chen X. Y. Zhang J. Zhu J. S. (2019). The role of m(6)A RNA methylation in human cancer. Mol. cancer 18, 103. 10.1186/s12943-019-1033-z 31142332 PMC6540575

[B9] Chung J. Y. Lee S. J. Lee H. J. Bong J. B. Lee C. H. Shin B. S. (2021). Aconitine neurotoxicity according to administration methods. J. Clin. Med. 10, 2149. 10.3390/jcm10102149 34065630 PMC8155921

[B10] Dominissini D. Moshitch-Moshkovitz S. Schwartz S. Salmon-Divon M. Ungar L. Osenberg S. (2012). Topology of the human and mouse m6A RNA methylomes revealed by m6A-seq. Nature 485, 201–206. 10.1038/nature11112 22575960

[B11] Dominissini D. Nachtergaele S. Moshitch-Moshkovitz S. Peer E. Kol N. Ben-Haim M. S. (2016). The dynamic N(1)-methyladenosine methylome in eukaryotic messenger RNA. Nature 530, 441–446. 10.1038/nature16998 26863196 PMC4842015

[B12] Erson-Bensan A. E. Begik O. (2017). m6A Modification and Implications for microRNAs. MicroRNA Shariqah, United Arab. Emir. 6, 97–101. 10.2174/2211536606666170511102219 28494721

[B13] Filomeni G. De Zio D. Cecconi F. (2015). Oxidative stress and autophagy: the clash between damage and metabolic needs. Cell death Differ. 22, 377–388. 10.1038/cdd.2014.150 25257172 PMC4326572

[B14] Hikino H. Murayama M. (1985). Mechanism of the antinociceptive action of mesaconitine: participation of brain stem and lumbar enlargement. Br. J. Pharmacol. 85, 575–580. 10.1111/j.1476-5381.1985.tb10551.x 3839708 PMC1916511

[B15] Hsu S. S. Liang W. Z. (2021). Cytotoxic effects of mesaconitine, the *Aconitum carmichaelii* debx bioactive compound, on HBEC-5i human brain microvascular endothelial cells: role of Ca^2+^ signaling-mediated pathway. Neurotox. Res. 39, 256–265. 10.1007/s12640-020-00249-2 32588354

[B16] Huang H. Weng H. Chen J. (2020). The biogenesis and precise control of RNA m(6)A methylation. Trends Genet. TIG 36, 44–52. 10.1016/j.tig.2019.10.011 31810533 PMC6925345

[B17] Huang Y.-P. Xia Y. Yang L. Wei J. Yang Y. I. Gao Y. Q. (2022). SPONGE: a GPU-accelerated molecular dynamics package with enhanced sampling and AI-driven algorithms. Chin. J. Chem. 40, 160–168. 10.1002/cjoc.202100456

[B18] Jiao X. Sherman B. T. Huang da W. Stephens R. Baseler M. W. Lane H. C. (2012). DAVID-WS: a stateful web service to facilitate gene/protein list analysis. Bioinformatics 28, 1805–1806. 10.1093/bioinformatics/bts251 22543366 PMC3381967

[B19] Jones J. D. Monroe J. Koutmou K. S. (2020). A molecular-level perspective on the frequency, distribution, and consequences of messenger RNA modifications. Wiley Interdiscip. Rev. RNA 11, e1586. 10.1002/wrna.1586 31960607 PMC8243748

[B20] Jumper J. Evans R. Pritzel A. Green T. Figurnov M. Ronneberger O. (2021). Highly accurate protein structure prediction with AlphaFold. Nature 596, 583–589. 10.1038/s41586-021-03819-2 34265844 PMC8371605

[B21] Kim D. Paggi J. M. Park C. Bennett C. Salzberg S. L. (2019). Graph-based genome alignment and genotyping with HISAT2 and HISAT-genotype. Nat. Biotechnol. 37, 907–915. 10.1038/s41587-019-0201-4 31375807 PMC7605509

[B23] Li X. Xie X. Gu Y. Zhang J. Song J. Cheng X. (2021). Fat mass and obesity-associated protein regulates tumorigenesis of arecoline-promoted human oral carcinoma. Cancer Med. 10, 6402–6415. 10.1002/cam4.4188 34378866 PMC8446412

[B24] Li Y. Ren J. Zhang Z. Weng Y. Zhang J. Zou X. (2023). Modification and expression of mRNA m6A in the lateral habenular of rats after long-term exposure to blue light during the sleep period. Genes 14, 143. 10.3390/genes14010143 36672884 PMC9859551

[B25] Liu X. Meng L. Li X. Li D. Liu Q. Chen Y. (2020). Regulation of FN1 degradation by the p62/SQSTM1-dependent autophagy-lysosome pathway in HNSCC. Int. J. oral Sci. 12, 34. 10.1038/s41368-020-00101-5 33318468 PMC7736930

[B26] Liu Y. Song R. Zhao L. Lu Z. Li Y. Zhan X. (2022). m6A demethylase ALKBH5 is required for antibacterial innate defense by intrinsic motivation of neutrophil migration. Signal Transduct. Target. Ther. 7, 194. 10.1038/s41392-022-01020-z 35764614 PMC9240034

[B27] Love M. I. Huber W. Anders S. (2014). Moderated estimation of fold change and dispersion for RNA-seq data with DESeq2. Genome Biol. 15, 550. 10.1186/s13059-014-0550-8 25516281 PMC4302049

[B28] Ma J. Song B. Wei Z. Huang D. Zhang Y. Su J. (2022). m5C-Atlas: a comprehensive database for decoding and annotating the 5-methylcytosine (m5C) epitranscriptome. Nucleic acids Res. 50, D196–d203. 10.1093/nar/gkab1075 34986603 PMC8728298

[B29] Maier J. A. Martinez C. Kasavajhala K. Wickstrom L. Hauser K. E. Simmerling C. (2015). ff14SB: improving the accuracy of protein side chain and backbone parameters from ff99SB. J. Chem. theory Comput. 11, 3696–3713. 10.1021/acs.jctc.5b00255 26574453 PMC4821407

[B30] Meng J. Cui X. Rao M. K. Chen Y. Huang Y. (2013). Exome-based analysis for RNA epigenome sequencing data. Bioinformatics 29, 1565–1567. 10.1093/bioinformatics/btt171 23589649 PMC3673212

[B31] Meyer K. D. Saletore Y. Zumbo P. Elemento O. Mason C. E. Jaffrey S. R. (2012). Comprehensive analysis of mRNA methylation reveals enrichment in 3' UTRs and near stop codons. Cell 149, 1635–1646. 10.1016/j.cell.2012.05.003 22608085 PMC3383396

[B32] Mitamura M. Boussery K. Horie S. Murayama T. Van de Voorde J. (2002a). Vasorelaxing effect of mesaconitine, an alkaloid from *Aconitum* japonicum, on rat small gastric artery: possible involvement of endothelium-derived hyperpolarizing factor. Jpn. J. Pharmacol. 89, 380–387. 10.1254/jjp.89.380 12233816

[B33] Mitamura M. Horie S. Sakaguchi M. Someya A. Tsuchiya S. Van de Voorde J. (2002b). Mesaconitine-induced relaxation in rat aorta: involvement of Ca^2+^ influx and nitric-oxide synthase in the endothelium. Eur. J. Pharmacol. 436, 217–225. 10.1016/s0014-2999(01)01623-5 11858801

[B34] Murayama M. Hikino H. (1985). Effect of cyclic AMP on mesaconitine-induced analgesia in mice. Eur. J. Pharmacol. 108, 19–23. 10.1016/0014-2999(85)90278-x 2984017

[B35] Murayama M. Ito T. Konno C. Hikino H. (1984). Mechanism of analgesic action of mesaconitine. I. Relationship between analgesic effect and central monoamines or opiate receptors. Eur. J. Pharmacol. 101, 29–36. 10.1016/0014-2999(84)90027-x 6086363

[B36] Muroi M. Kimura I. Kimura M. (1990). Blocking effects of hypaconitine and aconitine on nerve action potentials in phrenic nerve-diaphragm muscles of mice. Neuropharmacology 29, 567–572. 10.1016/0028-3908(90)90069-4 2385329

[B37] Nesterova Y. V. Povetieva T. N. Suslov N. I. Semenov A. A. Pushkarskiy S. V. (2011). Antidepressant activity of diterpene alkaloids of *Aconitum* baicalense Turcz. Bull. Exp. Biol. Med. 151, 425–428. 10.1007/s10517-011-1347-3 22448357

[B38] Ogura J. Mitamura M. Someya A. Shimamura K. Takayama H. Aimi N. (2004). Mesaconitine-induced relaxation in rat aorta: role of Na^+^/Ca^2+^ exchangers in endothelial cells. Eur. J. Pharmacol. 483, 139–146. 10.1016/j.ejphar.2003.10.022 14729101

[B39] Onur R. Bozdagi O. Ayata C. (1995). Effects of aconitine on neurotransmitter release in the rat neuromuscular junction. Neuropharmacology 34, 1139–1145. 10.1016/0028-3908(95)00050-g 8532184

[B40] Pertea M. Kim D. Pertea G. M. Leek J. T. Salzberg S. L. (2016). Transcript-level expression analysis of RNA-seq experiments with HISAT, StringTie and Ballgown. Nat. Protoc. 11, 1650–1667. 10.1038/nprot.2016.095 27560171 PMC5032908

[B41] Shan S. Shen Z. Song F. (2018). Autophagy and acetaminophen-induced hepatotoxicity. Archives Toxicol. 92, 2153–2161. 10.1007/s00204-018-2237-5 29876591

[B42] Shi H. Wei J. He C. (2019). Where, when, and how: context-dependent functions of RNA methylation writers, readers, and erasers. Mol. Cell 74, 640–650. 10.1016/j.molcel.2019.04.025 31100245 PMC6527355

[B43] Singhuber J. Zhu M. Prinz S. Kopp B. (2009). *Aconitum* in traditional Chinese medicine: a valuable drug or an unpredictable risk? J. Ethnopharmacol. 126, 18–30. 10.1016/j.jep.2009.07.031 19651200

[B44] Song B. Chen K. Tang Y. Wei Z. Su J. de Magalhães J. P. (2021). ConsRM: collection and large-scale prediction of the evolutionarily conserved RNA methylation sites, with implications for the functional epitranscriptome. Briefings Bioinforma. 22, bbab088. 10.1093/bib/bbab088 33993206

[B45] Song B. Huang D. Zhang Y. Wei Z. Su J. Pedro de Magalhães J. (2023a). m6A-TSHub: unveiling the context-specific m6A methylation and m6A-affecting mutations in 23 human tissues. Genomics, proteomics Bioinforma. 21, 678–694. 10.1016/j.gpb.2022.09.001 PMC10787194 36096444

[B46] Song B. Tang Y. Chen K. Wei Z. Rong R. Lu Z. (2020). m7GHub: deciphering the location, regulation and pathogenesis of internal mRNA N7-methylguanosine (m7G) sites in human. Bioinformatics 36, 3528–3536. 10.1093/bioinformatics/btaa178 32163126

[B47] Song B. Wang X. Liang Z. Ma J. Huang D. Wang Y. (2023b). RMDisease V2.0: an updated database of genetic variants that affect RNA modifications with disease and trait implication. Nucleic acids Res. 51, D1388–d1396. 10.1093/nar/gkac750 36062570 PMC9825452

[B48] Sun B. Zhang M. Zhang Q. Ma K. Li H. Li F. (2014). Metabonomics study of the effects of pretreatment with glycyrrhetinic acid on mesaconitine-induced toxicity in rats. J. Ethnopharmacol. 154, 839–846. 10.1016/j.jep.2014.05.010 24846827

[B49] Tang Y. Chen K. Song B. Ma J. Wu X. Xu Q. (2021). m6A-Atlas: a comprehensive knowledgebase for unraveling the N6-methyladenosine (m6A) epitranscriptome. Nucleic acids Res. 49, D134–d143. 10.1093/nar/gkaa692 32821938 PMC7779050

[B50] van Hameren G. Campbell G. Deck M. Berthelot J. Gautier B. Quintana P. (2019). *In vivo* real-time dynamics of ATP and ROS production in axonal mitochondria show decoupling in mouse models of peripheral neuropathies. Acta neuropathol. Commun. 7, 86. 10.1186/s40478-019-0740-4 31186069 PMC6558672

[B51] Wang L. Hui H. Agrawal K. Kang Y. Li N. Tang R. (2020). m6A RNA methyltransferases METTL3/14 regulate immune responses to anti-PD-1 therapy. EMBO J. 39, e104514. 10.15252/embj.2020104514 32964498 PMC7560214

[B52] Wang X. C. Jia Q. Z. Yu Y. L. Wang H. D. Guo H. C. Ma X. D. (2021a). Inhibition of the I(Na/K) and the activation of peak I(Na) contribute to the arrhythmogenic effects of aconitine and mesaconitine in Guinea pigs. Acta Pharmacol. Sin. 42, 218–229. 10.1038/s41401-020-0467-6 32747718 PMC8027609

[B53] Wang Y. Chen K. Wei Z. Coenen F. Su J. Meng J. (2021b). MetaTX: deciphering the distribution of mRNA-related features in the presence of isoform ambiguity, with applications in epitranscriptome analysis. Bioinformatics 37, 1285–1291. 10.1093/bioinformatics/btaa938 33135046

[B54] Wu Y. Bao W. Ren J. Li C. Chen M. Zhang D. (2022a). Integrated profiles of transcriptome and mRNA m6A modification reveal the intestinal cytotoxicity of aflatoxin B1 on HCT116 cells. Genes 14, 79. 10.3390/genes14010079 36672820 PMC9858580

[B55] Wu Y. Chen X. Bao W. Hong X. Li C. Lu J. (2022b). Effect of humantenine on mRNA m6A modification and expression in human colon cancer cell line HCT116. Genes 13, 781. 10.3390/genes13050781 35627166 PMC9140730

[B56] Ye H. Chen C. Wu H. Zheng K. Martín-Adrados B. Caparros E. (2022). Genetic and pharmacological inhibition of XBP1 protects against APAP hepatotoxicity through the activation of autophagy. Cell death Dis. 13, 143. 10.1038/s41419-022-04580-8 35145060 PMC8831621

[B57] Ye L. Gao S. Feng Q. Liu W. Yang Z. Hu M. (2012). Development and validation of a highly sensitive UPLC-MS/MS method for simultaneous determination of aconitine, mesaconitine, hypaconitine, and five of their metabolites in rat blood and its application to a pharmacokinetics study of aconitine, mesaconitine, and hypaconitine. Xenobiotica; fate foreign Compd. Biol. Syst. 42, 518–525. 10.3109/00498254.2011.641608 22188409

[B58] Ye Q. Liu H. Fang C. Liu Y. Liu X. Liu J. (2021). Cardiotoxicity evaluation and comparison of diterpene alkaloids on zebrafish. Drug Chem. Toxicol. 44, 294–301. 10.1080/01480545.2019.1586916 30895830

[B59] Yin R. Chang J. Li Y. Gao Z. Qiu Q. Wang Q. (2022). Differential m6A RNA landscapes across hematopoiesis reveal a role for IGF2BP2 in preserving hematopoietic stem cell function. Cell stem Cell 29, 149–159.e7. 10.1016/j.stem.2021.09.014 34678169

[B60] Zhang Y. Jiang J. Ma J. Wei Z. Wang Y. Song B. (2023). DirectRMDB: a database of post-transcriptional RNA modifications unveiled from direct RNA sequencing technology. Nucleic acids Res. 51, D106–d116. 10.1093/nar/gkac1061 36382409 PMC9825532

[B61] Zhao J. Zhu A. Sun Y. Zhang W. Zhang T. Gao Y. (2020a). Beneficial effects of sappanone A on lifespan and thermotolerance in *Caenorhabditis elegans* . Eur. J. Pharmacol. 888, 173558. 10.1016/j.ejphar.2020.173558 32941928

[B62] Zhao L. Sun Z. Yang L. Cui R. Yang W. Li B. (2020b). Neuropharmacological effects of aconiti lateralis radix praeparata. Clin. Exp. Pharmacol. physiology 47, 531–542. 10.1111/1440-1681.13228 31837236

[B63] Zhao Y. Shi Y. Shen H. Xie W. (2020c). m6A-binding proteins: the emerging crucial performers in epigenetics. J. Hematol. Oncol. 13, 35. 10.1186/s13045-020-00872-8 32276589 PMC7146974

[B64] Zhao Z. Zeng J. Guo Q. Pu K. Yang Y. Chen N. (2021). Berberine suppresses stemness and tumorigenicity of colorectal cancer stem-like cells by inhibiting m6A methylation. Front. Oncol. 11, 775418. 10.3389/fonc.2021.775418 34869024 PMC8634032

[B65] Zhou G. Tang L. Zhou X. Wang T. Kou Z. Wang Z. (2015). A review on phytochemistry and pharmacological activities of the processed lateral root of *Aconitum carmichaelii* Debeaux. J. Ethnopharmacol. 160, 173–193. 10.1016/j.jep.2014.11.043 25479152

[B66] Zhou J. Li X. Y. Liu Y. J. Feng J. Wu Y. Shen H. M. (2022). Full-coverage regulations of autophagy by ROS: from induction to maturation. Autophagy 18, 1240–1255. 10.1080/15548627.2021.1984656 34662529 PMC9225210

[B67] Zhu A. Sun Y. Zhong Q. Yang J. Zhang T. Zhao J. (2019). Effect of euphorbia factor L1 on oxidative stress, apoptosis, and autophagy in human gastric epithelial cells. Phytomedicine Int. J. phytotherapy Phytopharm. 64, 152929. 10.1016/j.phymed.2019.152929 31454650

[B68] Zong S. Wu Y. Li W. You Q. Peng Q. Wang C. (2023). SARS-CoV-2 Nsp8 induces mitophagy by damaging mitochondria. Virol. Sin. 38, 520–530. 10.1016/j.virs.2023.05.003 37156297 PMC10163945

[B69] Zorov D. B. Juhaszova M. Sollott S. J. (2014). Mitochondrial reactive oxygen species (ROS) and ROS-induced ROS release. Physiol. Rev. 94, 909–950. 10.1152/physrev.00026.2013 24987008 PMC4101632

